# Parasitism and alternative reproductive tactics in Northern chamois

**DOI:** 10.1002/ece3.5427

**Published:** 2019-07-05

**Authors:** Luca Corlatti, Chiara Lorenzetti, Bruno Bassano

**Affiliations:** ^1^ Wildlife Ecology and Management University of Freiburg Freiburg Germany; ^2^ Department of Veterinary Science University of Turin Grugliasco Italy; ^3^ Alpine Wildlife Research Centre Gran Paradiso National Park Valsavarenche Italy

**Keywords:** generalized additive mixed model, hormones, lungworms, reproduction, ungulates

## Abstract

Alternative reproductive tactics (ARTs), discrete phenotypic variations evolved to maximize fitness, may entail different cost‐benefit trade‐offs. In large mammals, differences in costs associated with ARTs—including energy expenditure and parasite infection—are typically greatest during the breeding season. Nonetheless, physiological and behavioral differences between ARTs can manifest throughout the year, possibly involving costs that may contribute to maintain ARTs within populations. Using the number of nematode larvae per gram of feces (LPG) as a proxy, we explored the temporal changes in lung parasite infection in territorial and nonterritorial male chamois *Rupicapra* in the Gran Paradiso National Park (Italy), between 2011 and 2012. We aimed to identify which tactic‐specific physiological and behavioral features (including age, hormonal levels, inter‐ and intrasexual interactions, and space use) or climatic factors (temperature and precipitation) best explained yearly variation in parasite infection within and between ARTs. Generalized additive mixed models showed that the fecal larval counts of lung nematodes underwent strong temporal changes in both male types. Differences between ARTs (with higher LPG values in territorial than nonterritorial males) were greatest during the rut and—to a lesser extent—in spring, respectively, at the peak and at the onset of territoriality. The difference in LPG between tactics was largely explained by the greater levels of hormone metabolites in territorial males during the rut. The other variables did not contribute significantly to explain the different shedding of larvae within and between ARTs. Our analysis suggests that different values of LPG between territorial and nonterritorial males are largely a result of tactic‐specific differences in the secretion of hormone metabolites, but only during the rut. To clarify whether rut‐related parasitism contributes to the maintenance of ARTs, tactic‐specific life history trade‐offs, for example, between reproduction and parasite‐related mortality, must be investigated.

## 1 INTRODUCTION

Polygynous mating systems are often associated with the development of alternative male reproductive tactics (ARTs), polymorphic traits that occur within species or populations and are selected to maximize fitness (Taborsky, Oliveira, & Brockmann, 2008). ARTs may manifest through a variety of phenotypes, including differences in color morph, body size, or behavior (Taborsky et al., 2008). In mammals, ARTs typically imply variations in mate guarding behavior by males, such as defending individual females, harems, or territories (Clutton‐Brock, 1989).

The mechanisms explaining the evolution of ARTs may entail frequency‐dependent or condition‐dependent selection (Gross, 1996) or a combination of the two (Plaistow, Johnstone, Colegrave, & Spencer, 2004). More generally, the understanding of processes favoring the evolution of ARTs may benefit from the investigation of the costs and benefits associated with different phenotypes (Engqvist & Taborsky, 2016; Plaistow et al., 2004). In large mammals, the costs of parasitism in alternative male types received some attention (Corlatti et al., 2012; Pelletier, Page, Ostiguy, & Festa‐Bianchet, 2005).

Differences in parasite infection between ARTs are largely due to tactics‐specific features expressed during the breeding season, including variations in mating effort and testosterone and cortisol levels. Mating effort and hormonal levels, for example, are typically greater in dominant than in subordinate individuals during the rut (Mooring et al., 2006; Pelletier et al., 2005), and they may increase exposure and susceptibility to parasite infection through increased encounter rates with conspecifics, energy depletion, and immunosuppression (Habig, Doellman, Woods, Olansen, & Archie, 2018). In contrast, parasite infection relative to ARTs outside of the breeding season has received little attention in mammals. Mating types, however, may express different behavioral and/or physiological patterns throughout the year. These patterns may be associated with differences in parasite infection, which in turn may contribute to the maintenance of ARTs within populations. Year‐round, tactic‐specific exposure and susceptibility to parasites, for example, may arise from different patterns of space use between male types, as infection probability may decrease with increasing elevation (Zanet et al., 2017) and home range size (Enzewa, 2004). Furthermore, infection probability can be indirectly influenced by variables that affect larval development such as temperature and precipitation (Taylor, Coop, & Wall, 2008).

In Northern chamois *Rupicapra rupicapra*, a mountain ungulate widely distributed in Europe and the Near East (Corlatti, Lorenzini, & Lovari, 2011), at least two ARTs occur, territorial and nonterritorial males (Corlatti et al., 2012; von Hardenberg, Bassano, Peracino, & Lovari, 2000). During the November rut, the higher mating success of territorial males is traded‐off against an increase in fecal egg/larval counts, compared to nonterritorial males (Corlatti et al., 2012). Outside of the rut, however, no information is available about tactic‐specific changes in parasite infection in chamois and, more generally, in mountain ungulates. The onset of territoriality in chamois occurs in spring, well before the rutting season (von Hardenberg et al., 2000). Thus, over large part of the year, male types may express different physiological and behavioral features, such as different hormonal levels (Corlatti, Palme, & Lovari, 2014) and patterns of space use, with territorial males occupying smaller home ranges at lower elevations compared to nonterritorial males (Corlatti, Bassano, Valencak, & Lovari, 2013; von Hardenberg et al., 2000). In turn, different patterns of parasite infection may occur in alternative phenotypes throughout the year.

In this study, we first aimed to model ART‐specific temporal patterns of lungworm larvae in feces. Data available on chamois suggest a bimodal spring‐autumn pattern of shedding of larvae (Stefancíková, Chovancová, Hájek, Dudinák, & Snábel, 2011). However, we also expected territorial males to have greater fecal larval counts than nonterritorial ones during the onset and the peak of territoriality (owing to their dominant status, *cf*. Corlatti et al., 2012) as well as during summer (owing to their spatial behavior, “risk‐prone” to parasite infection). Second, we aimed to identify which physiological and behavioral features best explained the differences in proxies of lung parasite infection within and between tactics over the year. To this end, we investigated how the modeled tactic‐specific temporal pattern of larval counts changed, after controlling for the effects of several internal and external variables—including age, hormonal levels, inter‐ and intrasexual interactions, space use, temperature, and precipitation. We expected increasing frequencies of inter‐ and intrasexual interactions and increasing hormone metabolite levels to positively affect fecal larval counts in male chamois (Corlatti et al., 2012; Hoby, Schwarzenberger, Doherr, Robert, & Walzer, 2006). Given the greater level of interactions and hormone metabolites in territorial than nonterritorial males during the rut (*cf*. Corlatti et al., 2012; Corlatti et al., 2014), the inclusion of these variables in the model should reduce the between‐tactic difference in larval counts in autumn. Conversely, we expected increasing home range size and elevation to reduce larval counts. In turn, different spatial behaviors of male types in summer should reduce the modeled between‐tactic difference in fecal larval counts during the warm periods. Finally, we expected other parameters to associate positively (age and precipitation) or negatively (temperature) with the shedding of larvae (Taylor et al., 2008). If selected, these variables should explain the within‐tactic changes, rather than the between‐tactic difference in larval counts, as we expected them to have similar effects on both ARTs. Their inclusion in the model should thus flatten the temporal changes within both male types, rather than reduce the difference between them.

## 2 MATERIALS AND METHODS

### 2.1 Study area and population

The upper Orco Valley, a 10 km^2^ area between 1,800 and 3,000 m a.s.l. within the Gran Paradiso National Park (Western Italian Alps, 45°26′30″N, 7°08′30″E), has a continental climate with mean yearly rainfall of about 1,000 mm and mean temperatures between −4°C in winter and 13°C in summer. The study population consisted of twenty‐two adult males darted by the personnel of the Park and equipped with GPS (Global Positioning System) collars. Further details about the study area and population are available in Corlatti et al. (2012), Corlatti et al. (2013), and Corlatti et al. (2014). Males were classified as territorial (*n* = 10) and nonterritorial (*n* = 12), following Corlatti et al. (2012).

The distinction between male types was based on the cluster analysis of behavioral patterns and space use during the mating season 2011, for which several hours of observation were available (with the exception of two individuals captured in 2012, *cf*. Table 1). We assumed that territorial males would have higher site fidelity and win more intrasexual interactions than nonterritorial males, and that males did not change mating tactic between the two years of study (animals sampled over the two mating seasons showed similar values for both home range size and behavioral patterns, L. Corlatti own data). Specifically, for each individual, the home range (90% fixed kernel) was calculated using GPS locations with at least four satellites and Dilution of Precision values <10 (Lewis, Rachlow, Garton, & Vierling, 2007), and individual tracks were kernel‐smoothed with the plug‐in bandwidth selector (“hpi”) of Wand and Jones (1995). The proportion of intrasexual interactions won was calculated using behavioral data recorded throughout the mating season, during hourly sessions of observations ad libitum (Altmann, 1974) on each individual. A male was considered as winner if the opponent was chased away or displayed submissive behaviors (details in Corlatti et al., 2012). These two parameters were combined in a matrix and multivariate hierarchical clustering (Everitt, Landau, Leese, & Stahl, 2011) and were conducted using the Mahalanobis distance (Mahalanobis, 1936). Males with small home ranges and high values of intrasexual interactions won were classified as territorial (Table 1). Further details of the male classification into territorial and nonterritorial are available in Corlatti et al. (2012) and in the Appendix S1 RMarkdown file.

**Table 1:** 90% fixed kernel density (90KDE) home range, proportion of intrasexual interactions won and mating behavior of adult male chamois (*n* = 22) resulting from cluster analysis (see Appendix S1) during the 2011 rut in Gran Paradiso National Park

Animal ID	90KDE (in ha)	Proportion of intrasexual interactions won	Mating behavior
M1	4.56	0.93	Territorial
M3	—	—	Nonterritorial^a^
M4	3.13	1.00	Territorial
M5	12.00	0.88	Territorial
M7	237.38	0.00	Nonterritorial
M8	16.63	0.00	Nonterritorial
M9	23.75	0.08	Nonterritorial
M11	7.81	1.00	Territorial
M12	13.06	0.38	Nonterritorial
M13	2.25	1.00	Territorial
M14	3.00	0.90	Territorial
M15	16.00	0.00	Nonterritorial
M16	5.75	1.00	Territorial
M17	4.63	1.00	Territorial
M18	16.94	0.50	Nonterritorial
M19	13.00	1.00	Territorial
M21	290.31	0.00	Nonterritorial
M22	75.50	0.46	Nonterritorial
M23	598.50	0.00	Nonterritorial
M24	8.75	0.25	Nonterritorial
M25	5.60	0.80	Territorial^b^
M26	6.40	0.00	Nonterritorial^b^

a M3 lacked data for the rutting season, and it was classified as “nonterritorial” based on the old age (13.5 years).

b Data for M25 and M26 were recorded during the 2012 rut.

### 2.2 Larval counts

Lung parasites causing bronchopulmonary strongylosis belong to the families Metastrongyloidae (genera *Metastrongylus* and *Dyctiocalus*) and Protostrongylidae (genera *Protostrongylus*, *Muellerius,* and *Cystocaulus*). In chamois, *Dictyocaulus*, *Protostrongylus*, *Cystocaulus,* and *Muellerius* are found most frequently (Taylor et al., 2008). They need an intermediate host to develop, typically a ground gasteropod of the genus *Helicella*, *Agriolimax,* or *Euparipha*, except for *Dictyocaulus* spp., whose cycle is direct. Infected chamois release L_1_ larvae with feces. L_1_ larvae penetrate in the gasteropod, where they develop into L_3_ and are accidentally ingested by chamois during foraging. Our analysis was based on counts of first‐stage fecal larvae (L_1_) of lung nematodes, without differentiating among genera. Every month between January 2011 and December 2012, we attempted to collect 1 fresh fecal sample per individual, by radio‐tracking marked chamois. Overall, 393 samples were collected (207 for territorial males, 186 for nonterritorial males; 17.9 samples per animal ±6.1 *SD*). Within 10 hr from sampling, a portion of each scat was stored in a plastic bag and was refrigerated for 1 week at + 4°C. The number of larvae in each sample was assessed following Thienpont, Rochette, and Vanparijs (1979). Two grams of feces was mixed with 28.0 ml of a zinc sulfate solution diluted at 33% to obtain a specific density of 1,200 kg/m^3^, and subsequently filtered with a strainer. We filled two chambers of the McMaster slide using a Pasteur pipette. The counting of larvae started 5 min after loading the slide using a light‐optical microscope at 100× magnification. The lung nematode larvae counted in each sample were multiplied by 50 to obtain the number of larvae per gram of feces (LPG), which was assumed to be a proxy of parasite infection. It is worth noting that coprological analyses quantifying the emission of endoparasites, per se, cannot assess adult parasite burdens. The accuracy of larval counts for diagnosing infection, in fact, may be limited by density‐dependent responses in parasite fertility, host control of parasite fertility through immune‐response or body conditions (Byrne, Fogarty, Mooney, Marples, & Holland, 2018). Results of coprological analyses, however, significantly and positively correlate to adult parasite burdens in wild species (Byrne et al., 2018; Gassó et al., 2015) and are often used as a proxy of parasite infection.

### 2.3 Predictors

Tactic‐specific temporal changes in LPG were assessed by regressing larval counts against the Julian date of collection, grouping by territorial and nonterritorial males. Variation in temporal changes was analyzed in relation to ART‐specific predictors (age, inter‐ and intrasexual interactions, hormonal levels, elevation, home range size), and climatic predictors (temperature, precipitation).

Age of chamois at sampling was estimated by counting horn rings during captures (Schröder & von Elsner‐Schack, 1984). Inter‐ and intrasexual interactions (i.e., courtship and aggressive behaviors, hereafter “interactions”) were investigated by recording individual activity budget data using focal sampling at 5‐min intervals (Altmann, 1974). Behavioral categories included foraging, lying down, moving, and standing interactions with males or females. Each animal for which we collected fecal samples was observed for a total of 2 hr (1 + 1 hr) within each month at different times of the day. Interactions were estimated by dividing the number of interactions with either sex by the total number of events (i.e., the sum of interactions and other behaviors). We assumed that, during the rut, interactions represented a proxy of mating effort.

Hormonal levels were assessed using a portion of each fecal sample, frozen at −20°C within 10 hr from sampling. Cortisol and androgen metabolite levels were measured by extracting 0.5 g of each well‐homogenized fecal sample with 5 ml aqueous methanol (80%) and using an 11‐oxoaetiocholanolone and a testosterone enzyme immunoassay. Details of the two EIAs, including cross‐reactions of the antibodies, are available in Möstl, Maggs, Schrötter, Besenfelder, and Palme (2002) and Palme and Möstl (1994), respectively. All samples were analyzed in duplicate, *cf*. Corlatti et al. (2014).

Spatial data were retrieved from GPS collars. Locations were collected every 11h, for an average amount of *ca*. 60 fixes/month/individual. For each animal, we calculated the mean elevation and the home range size in the month prior to fecal sample collection. Home range size for each animal was estimated using 90% kernel density (*cf*. Corlatti et al., 2012).

**Figure 1:**
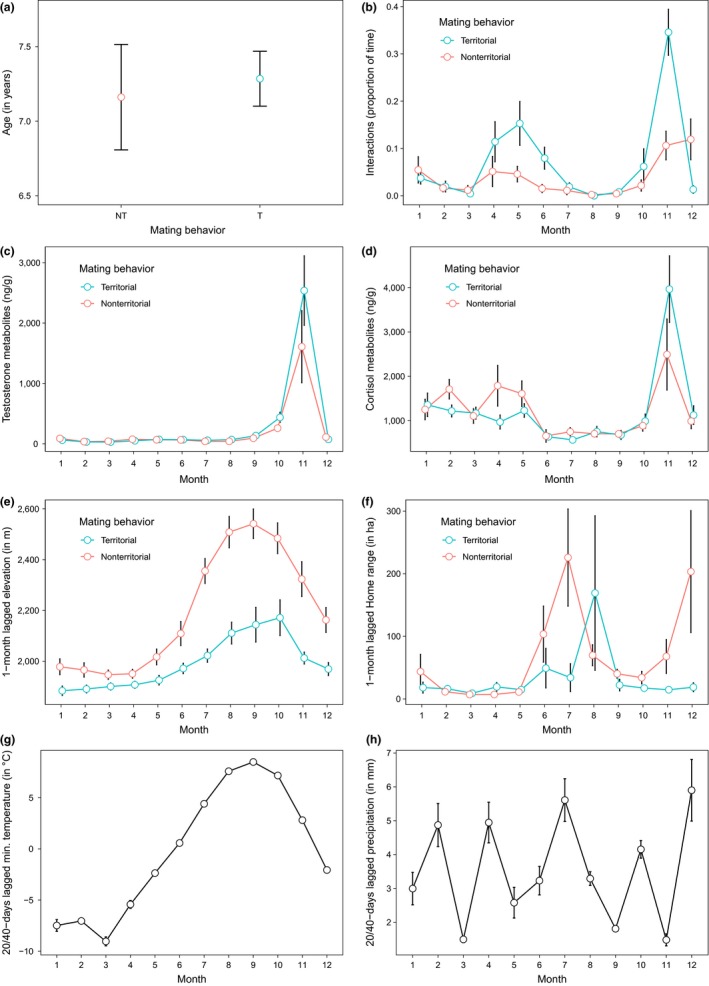
Tactic‐specific and/or monthly patterns of predictors used to model the temporal changes in fecal larval counts in territorial (T) and nonterritorial (NT) male chamois in Gran Paradiso National Park in 2011 and 2012. Age (a), proportion of time spent interacting with males or females (b), testosterone (c) and cortisol (d) metabolites, elevation (e), home range size (f), temperature (g) and precipitation (h). In panels b–h, the *x*‐axes report the month of fecal sampling. In panels e–h, the *y*‐axes report the values of elevation, home range size, temperature and precipitation recorded one month (e, f) or 20/40 days (g, h) before fecal sampling. Vertical bars represent 95% CI

Meteorological data were collected from a weather station in the study site (2,275 m a.s.l.). Mean minimum ambient temperature (in °C) and average precipitation (in mm) were calculated between 20 and 40 days prior to fecal sampling, as parasite eggs take about 10–20 days before an animal can be infested, and 15–20 days are needed before adult parasites can produce new eggs (Taylor et al., 2008). Tactic‐specific age differences and monthly patterns of testosterone and cortisol metabolites, interactions, lagged elevation, home range size, temperature, and precipitation are shown in Figure 1.

### 2.4 Statistical analyses

The effects of predictors on tactic‐specific temporal changes in LPG were investigated by modeling larval counts in sample*_i_*, first as a function of sampling date, then as a function of sampling date plus a predictor subset designated through model selection. All analyses were conducted with R 3.5.1 (R Core Team, 2018) in RStudio 1.1.456 (RStudio Team, 2016).

Parasite data are typically overdispersed and follow a negative binomial distribution, although in rare cases the Poisson distribution may be a good fit (Shaw, Grenfell, & Dobson, 1998). Thus, we first tested if LPG followed a Poisson distribution fitting a generalized linear model with the package “glmmTMB” (Brooks et al., 2017), where the Log of expected counts was presumed to be a linear function of all the predictors, plus a random intercept that incorporated the dependency among LPG values of the same individual. As some of the predictors (i.e., testosterone and cortisol metabolites, home ranges) showed unusually high values, they were Log_10_‐transformed prior to data analysis, to down‐weight the influence of extreme data points and improve the fit of the model (see RMarkdown file in Appendix S1). The function “overdisp” in the package “sjstats” (Lüdecke, 2018) returned a highly significant dispersion ratio for the Poisson model (Pearson's *χ* ^2^ = 99,728.804, *p*‐value < 0.001), and the function “zero_count” in “sjstats,” which tests for zero count overfitting, returned 117 observed zero counts versus 0 predicted zero counts. These tests indicated issues of overdispersion and zero inflation. Furthermore, we had to account for the correlation among LPG values that stemmed from multiple sampling of the same individuals over time, and for nonlinearity in some predictor–response relationships revealed by exploratory analysis.

The temporal change in LPG was thus investigated with a generalized additive mixed model (GAMM) with the package “gamlss” (Rigby & Stasinopoulos, 2005), assuming a negative binomial distribution and allowing for zero inflation. Our model was of the general form:

$$Y_{i}=\text{ZINB}\left(E\left[Y_{i}\right],\;\text{var}\left[Y_{i}\right]\right)$$

$$E\left[Y_{i}\right]=\mu_{i}\times \left(1-\pi_{i}\right)$$

$$\text{var}\left[Y_{i}\right]=\left(1-\pi_{i}\right)\times \left(\mu_{i}+\frac{\mu_{i}^{2}}{k}\right)+\mu_{i}^{2}\times \left(\pi_{i}^{2}+\pi_{i}\right)$$

$$\mu_{i}=e^{{\text{α}}_{1}+f_{1}\left(x_{1}\right)+\text{⋯}+f_{n}\left(x_{n}\right)+b_{i}}\;\text{and}\;\pi_{i}=\frac{e^{\alpha_{2}}}{1+e^{\alpha_{2}}}$$

where *μ_i_* and *π_i_* specified the link functions for the count data and the binomial data, *x_i_* were the selected predictors and *f_i_* the smoothing terms. For *μ_i_*, we assumed variation as a function of the intercept *α*_1_, different variables *x_i_*, plus a random intercept *b_i_* that was allowed to differ per individual. Variation in *π_i_* was assumed constant (i.e., as a function of the intercept *α*_2_).

We first modeled the plain temporal changes in LPG in territorial and nonterritorial males by fitting a “null” GAMM, where the Log of expected counts was assumed to be simply a function of Julian date grouped by mating behavior (territorial vs. nonterritorial males), using a penalized varying coefficient, plus the random term. So defined, this model can be thought of as a representation of the “pure” temporal changes in tactic‐specific LPG (i.e., the ART‐specific relationship between larval count and Julian date, without controlling for other variables except individual heterogeneity). Then, to investigate which variables best explained the tactic‐specific temporal patterns of LPG we added to the “null” model all the remaining predictors (except temperature, due to collinearity with Julian date), using penalized smoothers, and performed a stepwise selection to subset the variables that defined the “final” model, that is, the model with the lowest value of AIC (Akaike Information Criterion). The “final” model can be thought of as a representation of the “leftover” effect of tactic‐specific temporal change in LPG after controlling for the explanatory effect of the variable subset.

The comparison of the two GAMMs (“null” vs. “final”) thus allowed to depict if, and in which time of the year, some of the “pure” temporal change in LPG within and between ARTs could be explained by the selected predictors. Further details about the modeling procedure are available in the Appendix S1 RMarkdown file.

## 3 RESULTS

**Figure 2:**
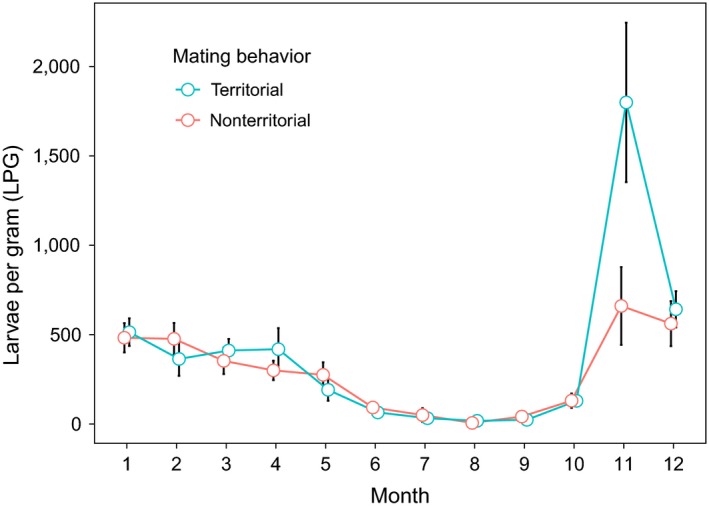
Tactic‐specific monthly patterns of lungworm larvae per gram of feces (LPG) in territorial and nonterritorial male chamois in Gran Paradiso National Park in 2011 and 2012. Vertical bars represent 95% CI

**Figure 3:**
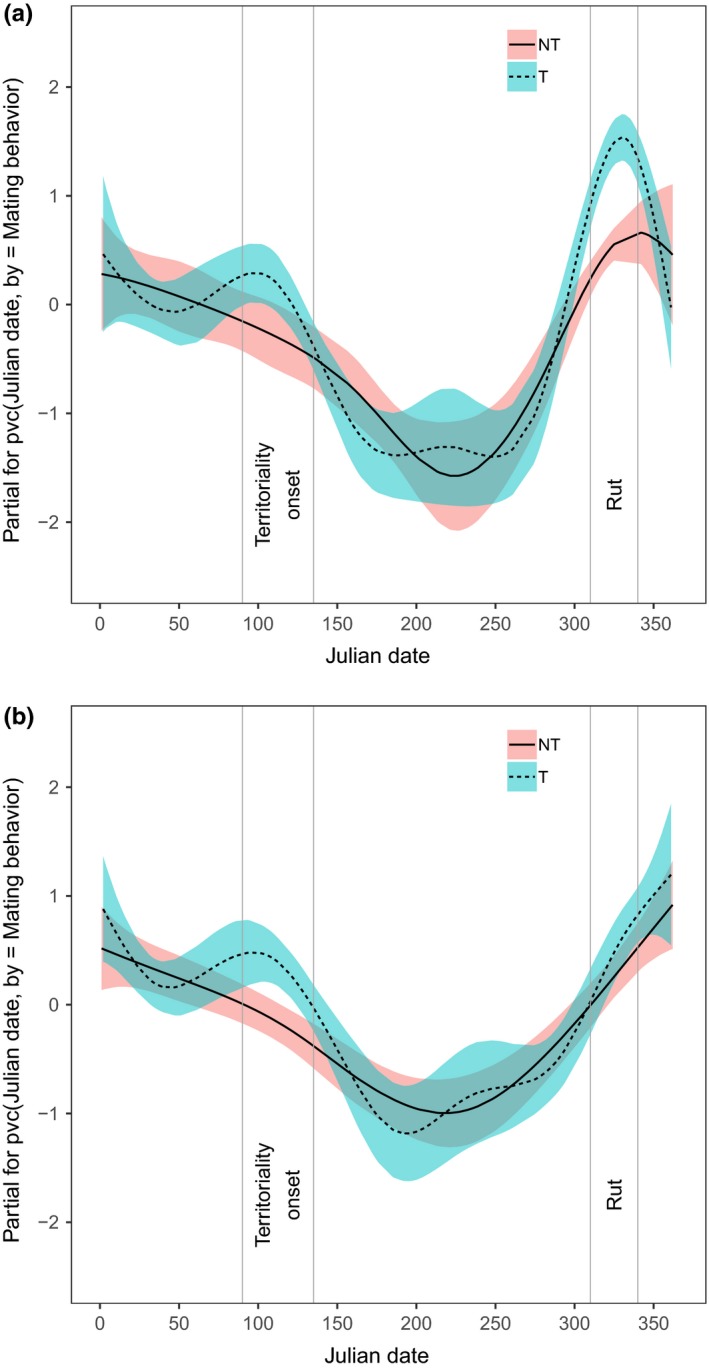
Marginal effects (with 95% CI) of Julian date on centered log‐values of larvae per gram in territorial (T) and nonterritorial (NT) male chamois in Gran Paradiso National Park in 2011 and 2012, obtained by fitting generalized additive mixed models (GAMMs). In (a), “pure” effect of time on the proxy of parasite infection. In (b), “leftover” effect of time after controlling for the explanatory effects of testosterone, cortisol, age, mating effort and precipitation. Vertical lines indicate the onset of territoriality (April to mid‐May) and the rutting period (early November–early December)

**Figure 4:**
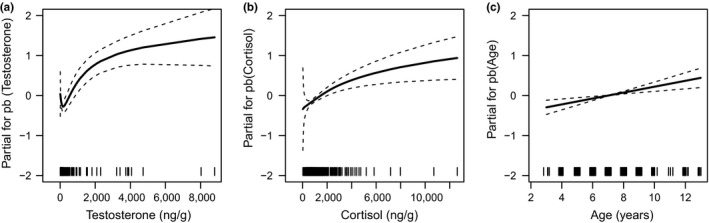
Marginal effects of (a) testosterone metabolites, (b) cortisol metabolites and (c) age on centered log‐values of larvae per gram in male chamois within the Gran Paradiso National Park in 2011 and 2012. Dashed lines represent 95% CI

Monthly changes in raw counts of LPG showed a bimodal pattern peaking in winter–spring and, especially, in autumn, when the difference in larval shedding between male types was greatest (territorial > nonterritorial, Figure 2). Outside of the territoriality periods, ARTs did not show major differences in larval counts (Figure 2). When the temporal change in tactic‐specific LPG was modeled using only Julian date grouped by mating behavior (territorial vs. nonterritorial) and individual heterogeneity (i.e., the “null” model), the bimodal pattern with differences between ARTs in spring and autumn was confirmed (Figure 3a). After including all predictors, the stepwise model selection returned a “final” model with Julian date grouped by mating behavior, age, interactions, Log_10_‐hormone metabolites, precipitation, and individual heterogeneity as predictors of LPG (Figure 3b). After adjusting the significance level for the maximum number of steps in the stepwise selection (Bonferroni's correction, *p*‐value threshold = 1.38 × 10^–7^; Zuur, Ieno, & Smith, 2007), LPG was positively related to hormone metabolites (Table 2) through nonlinear relationships (Figure 4a,b), and to age (Table 2) through a linear relationship (Figure 4c). Interactions and precipitation were not significant (Table 2). The “final” model returned a value of 0.57 for the generalized *R* ^2^ of Nagelkerke (1991). The same model fitted without random effect returned a generalized *R* ^2^ of 0.45, suggesting an important role of individual heterogeneity in the variation of LPG. The comparison of “null” and “final” model outputs showed that some of the “pure” temporal changes in LPG (Figure 3a) could be explained by our predictors, especially in territorial males, thus strongly reducing the difference in larval counts between ARTs, but only during the rut (Figure 3b).

**Table 2:** Estimates, standard errors (*SE*), *t*‐value, and *p*‐value of the predictors included in the generalized additive mixed model selected to explain temporal changes in fecal larval counts in male chamois in Gran Paradiso National Park in 2011 and 2012

	Estimate	*SE*	*t*‐Value	*p*‐Value
Intercept	−2.811	0.360	−7.816	6.46 × 10^–14^
pb(Age)	0.148	0.019	7.898	3.71 × 10^–14^
pb(Interactions)	0.301	0.339	0.908	0.365
pb(Log_10_‐Testosterone)	0.830	0.007	11.066	<2 × 10^–16^
pb(Log_10_‐Cortisol)	1.950	0.114	17.045	<2 × 10^–16^
pb(Precipitation)	0.060	0.011	5.269	2.41 × 10^–7^

Note

The table reports values for age (Age), inter‐ and intrasexual interactions (Interactions), log‐transformed testosterone and cortisol metabolites (Log_10_‐Testosterone, Log_10_‐Cortisol) and precipitation (Precipitation). “pb” refers to the penalized smoother used in “gamlss.”

## 4 DISCUSSION

The mean fitted values of LPG showed clear temporal fluctuations, with a winter/spring–autumn bimodal pattern and a peak during the rut, thus supporting previous results (Stefancíková et al., 2011). The difference in fecal larval counts between territorial and nonterritorial males was greatest in November, with higher values in the former than in the latter (*cf*. Corlatti et al., 2012). Part of the within‐ and between‐tactic temporal change was captured by hormone metabolites and age. Social interactions and precipitation were poorly informative, and no other external variable significantly affected fecal larval counts.

The difference between territorial and nonterritorial males in parasite infection strongly decreased during the rut, after controlling for the selected variables. This result suggests that the difference in LPG between male types in November may be primarily explained by steroid hormones, which are known to trigger immunosuppression (Hoby et al., 2006), as also suggested by our data. Previous studies on the same population showed that rutting territorial males have higher levels of cortisol and testosterone metabolites than nonterritorial ones (Corlatti et al., 2012; Corlatti et al., 2014; *cf*. Figure 1), and a stronger rut‐related immunosuppression (thus higher values of LPG) is expected in the former than in the latter (Corlatti et al., 2012). Conversely, the inclusion of hormone metabolites did not explain the greater values of LPG in territorial males in spring, at the onset of territoriality (von Hardenberg et al., 2000). This is not surprising, as in spring territorial males have lower levels of hormone metabolites than nonterritorial ones (Corlatti et al., 2014; *cf*. Figure 1).

The other variables included in the final model—age, interactions, and precipitation—did not explain different values of LPG between male types. For example, the positive relationship between age and larval counts, possibly related to senescence (Møller & De Lope, 1999), is likely to affect ARTs in similar ways, as territorial and nonterritorial male chamois do not differ in age (Corlatti et al., 2012; *cf*. Figure 1). Likewise, the seemingly positive effect of precipitation on LPG presumably affected shedding of larvae in similar ways in both male types, and its small effect size makes it a poorly informative parameter. Although mating effort may impose greater energetic constraints on dominant individuals than on subordinates—and thus hamper their immune defense, as observed in bighorn sheep *Ovis canadensis* (Pelletier et al., 2005)—the expected positive relationship between interactions and LPG was not supported, as the parameter estimates were too imprecise. Finally, the effects of ART‐specific spatial behaviors on larval counts (i.e., greater values of LPG in territorial than in nonterritorial males, owing to smaller home ranges at lower elevation) were dropped in the final model, suggesting that the influence of space use on the shedding of larvae was negligible. Similarly, no effect of precipitation was detected on larval counts.

The leftover temporal variation in LPG between and within ARTs, after controlling for the selected variables, needs further investigation. Factors affecting the relationships between the shedding of larvae and hosts (either intermediate or final), possibly including some unaccounted genetic and environmental feature such as temperature, which was excluded from our model due to collinearity with Julian date, might explain part of the variation (Taylor et al., 2008). However, unless these variables interact statistically with ARTs, they are more likely to explain within‐tactic, rather than between‐tactic differences. The role of other tactic‐specific variables on larval counts should thus be explored, possibly extending the investigation both temporally and geographically, to reduce sampling limitations.

With the exception of autumn and—to a much lesser extent—spring, territorial, and nonterritorial male chamois did not show major differences in parasite infection. Whether these short‐term differences are sufficient to impose different costs on alternative male types remains unclear. It should be pointed out that, in principle, the rapid temporal variations described in this study may result from changes in parasite fertility, rather than changes in infection intensity. However, our coprological analysis should reflect patterns of infection (*cf*. Byrne et al., 2018) and thus provide a proxy of the costs associated with different alternative reproductive tactics. A recent study, however, suggested that males may have developed natural mechanisms to compensate for higher parasite infection during the rut (Oliver‐Guimerá et al., 2017). If so, ARTs may not suffer different costs of parasitism.

More generally, a pattern of coevolution between parasites and hosts may occur, so that the development of behavioral strategies that minimize the impact of parasites may favor host populations. In this respect, host strategies that allow to control the infection may be beneficial not only to the hosts, but also to the parasites, through the maintenance of viable host populations (*cf*. the beneficial effect of sexual segregation in Alpine ibex *Capra ibex* interacting with parasites, Ferrari, Rosà, Lanfranchi, & Ruckstuhl, 2010). Whether a coevolution between chamois ARTs and parasite infection exists, so that the maintenance of alternative tactics would minimize the impact of parasites on both host types, however, is not known. Furthermore, Corlatti et al. (2012) suggested that—beside parasite infection—several other factors may concur to maintain ARTs, such as greater risks of injuries and greater consumption of fat reserves in territorial than in nonterritorial males. Our analysis did not distinguish among genera, and to our knowledge, no information is available on different pathogenicity of lung parasites in chamois. However, parasites of the families Metastrongyloidae and Protostrongylidae may be expected to have different temporal dynamics, thus possibly different effects on chamois life‐history traits depending on territoriality‐mediated exposure.

To shed light on the potential mechanisms linking parasitism and maintenance of ARTs within the same population, ART‐specific data on reproductive success and parasite‐related mortalities are needed (*cf*. Corlatti et al., 2012). In this respect, multievent capture–mark–recapture models may be particularly suited to estimate mortality probabilities by different sources (e.g., different parasite infections vs. other causes) while accounting for changes in the status of individuals (e.g., age‐dependent changes between territorial and nonterritorial behavior or vice‐versa), different detection probability among marks (e.g., optical tags, functioning/ nonfunctioning GPS collars), as well as for uncertainty in the causes of death of recovered carcasses (Pradel, 2005).

In summary, differences between territorial and nonterritorial male chamois in parasite infection mainly occur during the mating season and are largely explained by the combined effect of cortisol and testosterone metabolites. During the rest of the year, ARTs did not show major differences in larval counts. Some leftover variation in the temporal pattern of LPG within and between ARTs, however, still needs to be explained.

## CONFLICT OF INTERESTS

We have no competing interests.

## AUTHOR CONTRIBUTION

L.C. conceived the idea, collected data and did the statistical analyses. C.L. helped in data collection and in the analysis of fecal larval count. L.C. wrote the manuscript, with support from B.B., who also supervised all stages of this work.

## Data Availability

Data used in this analysis are available at Dryad Digital Repository: https://doi.org/10.5061/dryad.390n07q.
