# Supplementary material for: Parasitism and alternative reproductive tactics in Northern chamois
Source: Ecol Evol. 2019 Jul 5;9(15):8749–58. doi: 10.1002/ece3.5427 (PMC6686307; doi:10.1002/ece3.5427)
Supplement: Supplementary file 1 [file ECE3-9-8749-s001.pdf]

Supplementary file 1

Parasitism and alternative reproductive tactics in Northern chamois

Corlatti, L., Lorenzetti, C., Bassano, B.

RMarkdown

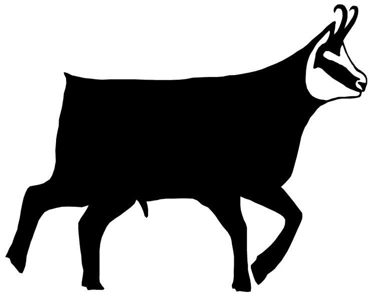

Content

Introduction
Territorial *vs.* nonterritorial males
1. Create dataframe
2. Cluster analysis
Data analysis
3. Import data
4. Inspect data
   2.1 Linearity of relationships and collinearity issues
   2.2 Overdispersion and zero-inflation.
5. Modelling
   5.1 Model fitting
   5.2 Model diagnostic
6. Plot model results
   6.1 Fig. 3
   6.2 Fig. 4
References

Introduction

Alternative reproductive tactics (ARTs), discrete phenotypic variations evolved to maximize fitness, may entail different trade-offs between benefits and costs. In large mammals, costs associated with the expression of ARTs are typically greatest during the breeding season. Nonetheless, physiological and behavioural differences between ARTs can manifest throughout the year, and they may be expected to entail costs that may favour the maintenance of alternative types within populations. Using larval counts from faeces as a proxy, we explored the temporal changes in lung parasite infection in territorial and nonterritorial male chamois *Rupicapra rupicapra* in the Gran Paradiso National Park, between 2011 and 2012. We aimed to identify if tactic-specific physiological and behavioural features (including age, hormonal levels, inter- and intrasexual interactions and space use) or climatic factors (temperature and precipitation) concur to explain the yearly variation in parasite infection within and between ARTs.

Territorial *vs.* nonterritorial males

1. Create dataframe

We first create a dataframe that includes ID of individuals (ID), ratio of interactions won during the rut (ratio), and home range size during the rut calculated using 90% Kernel smoothing (ks90).

```
ID <- c("M1","M4","M5","M7","M8","M9","M11","M12","M13","M14","M15","M16","M17","M18","M19","M21","M22","M23",
        "M24","M25","M26")
ratio <- c(0.93,1.00,0.88,0.00,0.00,0.08,1.00,0.38,1.00,0.90,0.00,1.00,1.00,0.50,1.00,0.00,0.46,0.00,
           0.25,0.75,0.00)
ks90 <- c(4.56,3.13,12.00,237.38,16.63,23.75,7.81,13.06,2.25,3.00,16.00,5.75,4.63,16.94,13.00,290.31,75.50,
          598.50,8.75,5.60,6.40)

cluster <- as.data.frame(cbind(ID,ratio,ks90))

fvars <- c(1) # defines the positions of factor variables
for (i in fvars){
  cluster[,i]<-as.factor(cluster[,i])
}

nvars <- c(2,3) # defines the positions of numeric variables
for (i in nvars){
  cluster[,i]<-as.numeric(as.character(cluster[,i]))
}

head(cluster)
#       ID      ratio   ks90
# 1     M1      0.93    4.56
# 2     M4      1.00    3.13
# 3     M5      0.88    12.00
# 4     M7      0.00    237.38
# 5     M8      0.00    16.63
# 6     M9      0.08    23.75

summary(cluster)
   ID           ratio            ks90
 # M1  : 1      Min.   :0.0000   Min.   :  2.25
 # M11 : 1      1st Qu.:0.0000   1st Qu.:  5.60
 # M12 : 1      Median :0.5000   Median : 12.00
 # M13 : 1      Mean   :0.5324   Mean   : 65.00
 # M14 : 1      3rd Qu.:1.0000   3rd Qu.: 16.94
 # M15 : 1      Max.   :1.0000   Max.   :598.50
 # (Other):15
```

2. Cluster analysis

The distinction between male types was based on the cluster analysis of behavioural patterns and space use during the mating season, assuming that territorial males have higher site fidelity and win more intra-sexual interactions than nonterritorial males. These two parameters were combined in a matrix and multivariate hierarchical clustering (Everitt et al. 2011) was conducted using the Mahalanobis distance (Mahalanobis 1936).

```
library(stats)
dat <- cbind(cluster$ratio, cluster$ks90) # combine the target variables in a matrix

varcov <- matrix(c(var(cluster$ratio), cov(cluster$ratio,cluster$ks90), cov(cluster$ratio,cluster$ks90),
                 var(cluster$ks90)), nrow=2, ncol=2) # calculate the variance-covariance

dist <- mahalanobis(dat,dat[1,], varcov) # calculate the Mahalanobis distance on the data matrix

for(i in 2:length(cluster$ratio)) dist<-cbind(dist, mahalanobis(dat, dat[i,], varcov) )

dist <- as.dist(dist)

hc <- hclust(dist) # run hierarchical clustering on the Mahalanobis distance

hc$labels <- 1:21 # add observation numbers

label<-as.factor(cluster$ID) # create character string ("labels") with the ID of individuals

plot(hc, labels=label) # plot cluster
```

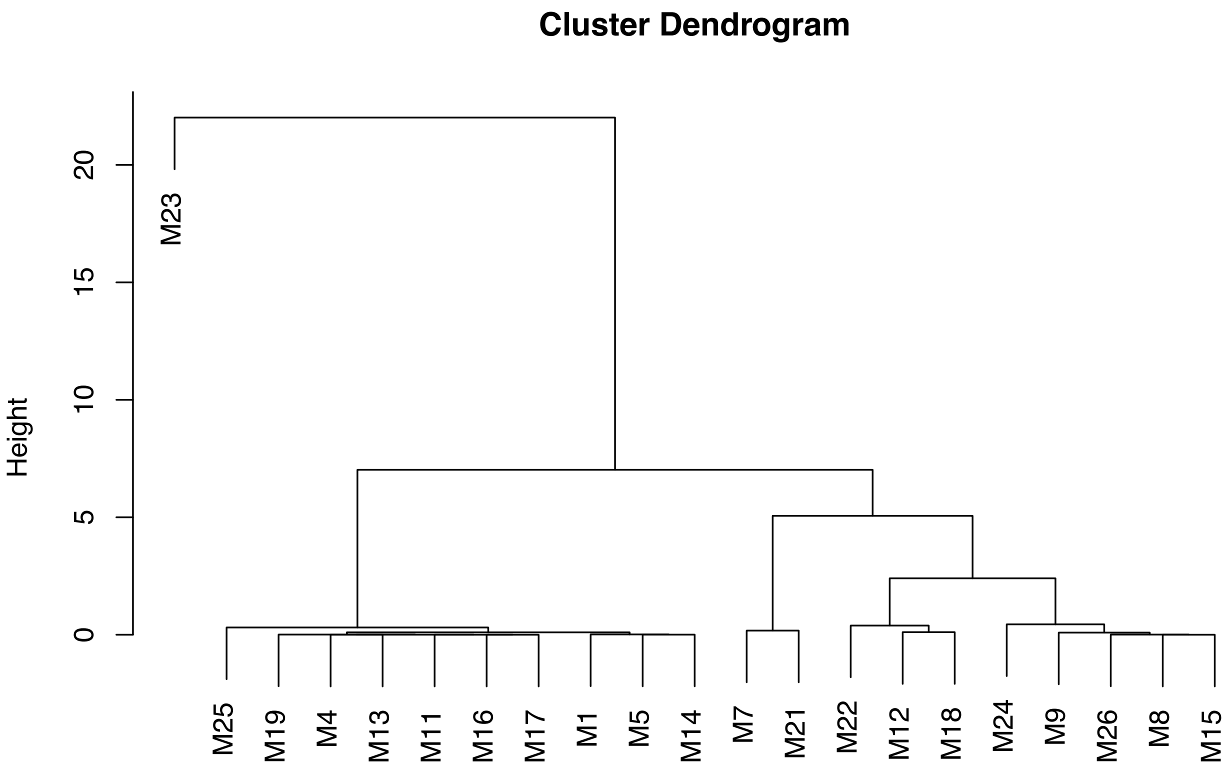

The cluster analysis indicates that 10 individuals with small home ranges and high ratio of interactions won (M25, M19, M4, M13, M11, M16, M17, M1, M5, M14) are grouped together ("territorial males"), whereas the other individuals are grouped in a different cluster (males with large home ranges and low ratio of interactions won, i.e., "nonterritorial"). One individual (M23) was classified as "nonterritorial" owing to the large home range (*ca.* 600 ha), while another individual (M3) was considered as nonterritorial owing to the advanced age at capture (13.5 years). See Table 1 in the article.

Data analysis

3. Import data

We first set up the working directory and load the data.

```
setwd("~/...") # Please choose a working directory
data.raw <- read.csv2("Corlatti_et_al_Parasites & ARTs chamois_Supporting Information_2.csv", header = T)
head(data.raw)
##   obs  id age m.beh     date lungworms testosterone cortisol   tmin prec   elev.lag kde90.lag interactions
## 1   1 M01   5     T 03/01/11      1150        54.41  1932.15 -10.50 1.90       1935      8.56     0.00
## 2   2 M16   6     T 03/01/11       450        50.82  2261.60 -10.50 1.90       1917      6.19     0.04
## 3   3 M17   6     T 04/01/11       100        56.63  1554.30 -10.50 1.90       1882      6.94     0.15
## 4   4 M03  12    NT 11/01/11      1100        46.82   660.55 -11.40 0.00       1785      8.94     0.00
## 5   5 M04   7     T 11/01/11       850        60.02  1806.20 -11.40 0.00       1774     14.75     0.00
## 6   6 M15   6    NT 11/01/11       250        47.10   807.95 -11.40 0.00       1886     19.81     0.04
##
```

Legend
obs = this is just a sequential list of numbers to indicate the position of the rows
id = identity of marked individuals (factor);
age = age (in years) of individuals at sampling (numeric);
m.beh = mating behaviour of individuals (T = territorial; NT = nonterritorial) (factor);
date = date of sampling (numeric);
lungworms = counts of lungworm larvae (n. / gr) (numeric);
testosterone = level of testosterone metabolites (ng / g) (numeric);
cortisol = level of cortisol metabolites (ng /g) (numeric);
tmin = average minimum ambient temperature (in °C) 20-40 days before sampling (numeric);
prec = average precipitation (in mm) 20-40 days before sampling (numeric);
elev.lag = average elevation of the animals in the month prior to fecal sampling (numeric);
kde90.lag = home range size (90% kernel density) in the month prior to fecal sampling (numeric);
interactions = inter- and intrasexual interactions measured through focal sampling at 5-minute intervals (numeric).

We check if all the variables have been specified correctly.

```
summary(data.raw)
##       obs               id           age         m.beh          date    
##  Min.   :  1.0   M04    : 24   Min.   : 3.000   NT:186   06/03/12:  7  
##  1st Qu.: 99.0   M05    : 24   1st Qu.: 6.000   T :207   01/02/11:  5  
##  Median :197.0   M11    : 24   Median : 7.000            05/07/12:  5  
##  Mean   :197.1   M13    : 24   Mean   : 7.226            07/09/12:  5  
##  3rd Qu.:295.0   M08    : 23   3rd Qu.: 8.000            08/03/11:  5  
##  Max.   :396.0   M12    : 23   Max.   :13.000            15/07/11:  5  
##                  (Other):251                             (Other) :361  
##    lungworms       testosterone    cortisol        tmin          prec     elev.lag      kde90.lag  interactions
##  Min.   :   0.0   14.74   :  2   724.90 :  3   -1.38   : 14   1.40    : 25   Min.   :1727   4.69   :  5   0.00 :260  
##  1st Qu.:   0.0   15.58   :  2   762.30 :  3   -6.86   : 14   10.84   : 18   1st Qu.:1917   4.00   :  4   0.04 : 49  
##  Median : 150.0   16.19   :  2   767.80 :  3   -7.30   : 14   0.00    : 17   Median :1999   4.63   :  4   0.08 : 23  
##  Mean   : 331.1   19.14   :  2   985.60 :  3   -12.14 : 12   0.57    : 14   Mean   :2092   4.88   :  4   0.15 : 11  
##  3rd Qu.: 400.0   19.60   :  2   1076.90:  2   -3.95   : 11   1.08    : 14   3rd Qu.:2162   5.00   :  4   0.12 : 10  
##  Max.   :6450.0   20.79   :  2   1087.90:  2   -6.45   : 11   9.16    : 14   Max.   :2978   5.75   :  4   0.23 :  7  
##                   (Other):381   (Other):377   (Other):317   (Other):291                  (Other):368   (Other):33  
##
```

We need to redefine the position of numeric and factor variables.

```
nvars <- c(3,6:13) # Define position of numeric variables
for (i in nvars){
  data.raw[,i]<-as.numeric(as.character(data.raw[,i]))
}

fvars <- c(1,2,4,5) # Define position of factor variables
for (i in fvars){
  data.raw[,i]<-as.factor(data.raw[,i])
}

data.parasites <- na.omit(data.raw)
summary(data.parasites)
```

```
##       obs             id           age          m.beh          date   
##  1      :  1   M04    : 24   Min.   : 3.000   NT:186   06/03/12:  7  
##  2      :  1   M05    : 24   1st Qu.: 6.000   T :207   01/02/11:  5  
##  3      :  1   M11    : 24   Median : 7.000            05/07/12:  5  
##  4      :  1   M13    : 24   Mean   : 7.226            07/09/12:  5  
##  5      :  1   M08    : 23   3rd Qu.: 8.000            08/03/11:  5  
##  6      :  1   M12    : 23   Max.   :13.000            15/07/11:  5  
##  (Other):387   (Other):251                             (Other) :361  
##    lungworms       testosterone        cortisol            tmin         
##  Min.   :   0.0   Min.   :   6.62   Min.   :   22.0   Min.   :-12.1400  
##  1st Qu.:   0.0   1st Qu.:  28.14   1st Qu.:  568.7   1st Qu.: -6.4500  
##  Median : 150.0   Median :  54.91   Median :  827.2   Median : -1.3800  
##  Mean   : 331.1   Mean   : 240.52   Mean   : 1201.3   Mean   : -0.2433  
##  3rd Qu.: 400.0   3rd Qu.: 114.18   3rd Qu.: 1412.4   3rd Qu.:  5.4000  
##  Max.   :6450.0   Max.   :8800.00   Max.   :12619.2   Max.   :  9.9500  
##                                                                         
##       prec           elev.lag      kde90.lag         interactions    
##  Min.   : 0.000   Min.   :1727   Min.   :   0.38   Min.   :0.00000  
##  1st Qu.: 1.400   1st Qu.:1917   1st Qu.:   5.75   1st Qu.:0.00000  
##  Median : 2.260   Median :1999   Median :  11.38   Median :0.00000  
##  Mean   : 3.561   Mean   :2092   Mean   :  49.37   Mean   :0.05351  
##  3rd Qu.: 4.630   3rd Qu.:2162   3rd Qu.:  27.00   3rd Qu.:0.04000  
##  Max.   :12.220   Max.   :2978   Max.   :2103.19   Max.   :0.69000  
## 
```

The dataset is OK.

Now we need to add the Julian date to the dataset, which will be used to test for the temporal effect.

```
library(lubridate)

data.parasites$POSIX.date <- as.POSIXlt(data.parasites$date, format = "%d/%m/%y")

data.parasites$julian.date <- (data.parasites$POSIX.date)$yday

summary(data.parasites)
##       obs             id           age          m.beh          date   
##  1      :  1   M04    : 24   Min.   : 3.000   NT:186   06/03/12:  7  
##  2      :  1   M05    : 24   1st Qu.: 6.000   T :207   01/02/11:  5  
##  3      :  1   M11    : 24   Median : 7.000            05/07/12:  5  
##  4      :  1   M13    : 24   Mean   : 7.226            07/09/12:  5  
##  5      :  1   M08    : 23   3rd Qu.: 8.000            08/03/11:  5  
##  6      :  1   M12    : 23   Max.   :13.000            15/07/11:  5  
##  (Other):387   (Other):251                             (Other) :361  
## 
##    lungworms       testosterone        cortisol            tmin         
##  Min.   :   0.0   Min.   :   6.62   Min.   :   22.0   Min.   :-12.1400  
##  1st Qu.:   0.0   1st Qu.:  28.14   1st Qu.:  568.7   1st Qu.: -6.4500  
##  Median : 150.0   Median :  54.91   Median :  827.2   Median : -1.3800  
##  Mean   : 331.1   Mean   : 240.52   Mean   : 1201.3   Mean   : -0.2433  
##  3rd Qu.: 400.0   3rd Qu.: 114.18   3rd Qu.: 1412.4   3rd Qu.:  5.4000  
##  Max.   :6450.0   Max.   :8800.00   Max.   :12619.2   Max.   :  9.9500  

##       prec           elev.lag      kde90.lag         interactions    
##  Min.   : 0.000   Min.   :1727   Min.   :   0.38   Min.   :0.00000  
##  1st Qu.: 1.400   1st Qu.:1917   1st Qu.:   5.75   1st Qu.:0.00000  
##  Median : 2.260   Median :1999   Median :  11.38   Median :0.00000  
##  Mean   : 3.561   Mean   :2092   Mean   :  49.37   Mean   :0.05351  
##  3rd Qu.: 4.630   3rd Qu.:2162   3rd Qu.:  27.00   3rd Qu.:0.04000  
##  Max.   :12.220   Max.   :2978   Max.   :2103.19   Max.   :0.69000  
## 
##    POSIX.date                   julian.date   
##  Min.   :2011-01-03 00:00:00   Min.   :  1.0  
##  1st Qu.:2011-06-24 00:00:00   1st Qu.: 81.0  
##  Median :2011-12-20 00:00:00   Median :178.0  
##  Mean   :2011-12-17 05:09:09   Mean   :175.6  
##  3rd Qu.:2012-06-10 00:00:00   3rd Qu.:270.0  
##  Max.   :2012-12-27 00:00:00   Max.   :362.0
```

4. Inspect data

4.1 Linearity of relationships and collinearity issues

```
library(PerformanceAnalytics)

chart.Correlation(data.parasites[, c(3,6:13,15)], histogram=TRUE, pch=19)
```

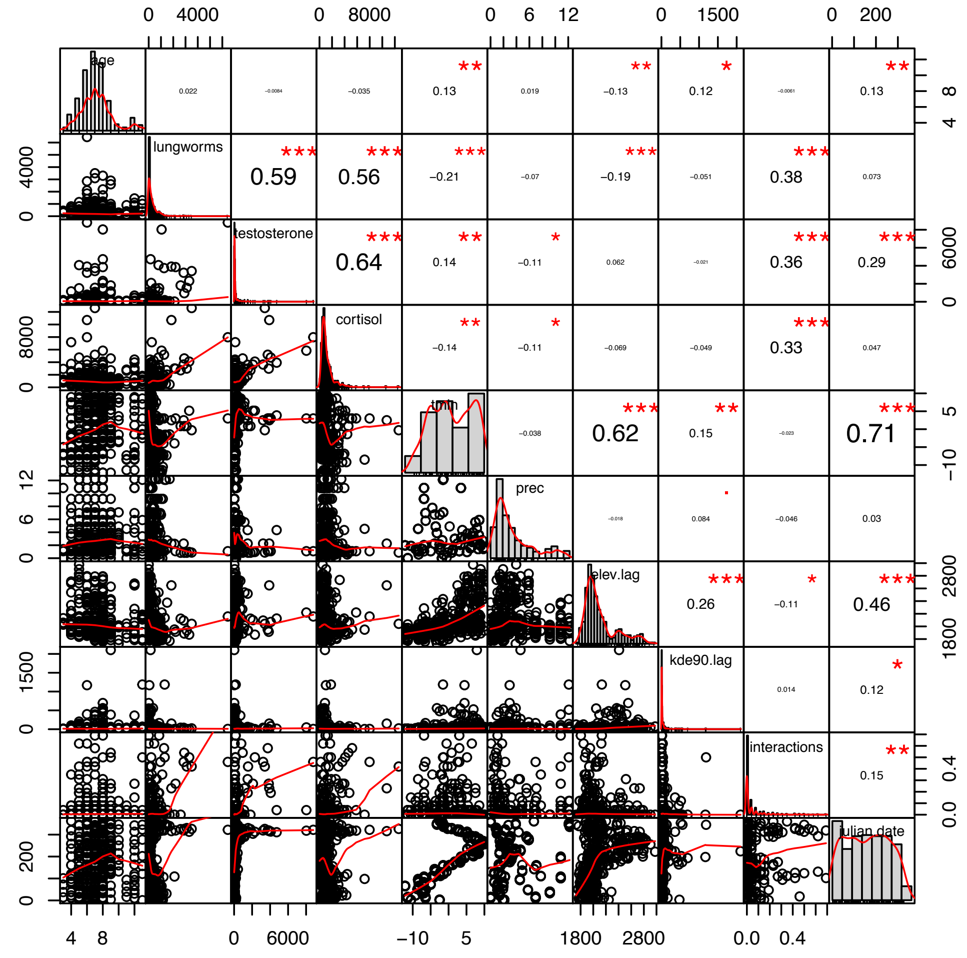

No major issue of collinearity is present, as correlation coefficients are < 0.7, except for julian.date~tmin. We note that there is a clear pattern in the julian.date~tmin panel. It shows a sinusoidal pattern reflecting the seasonal temperature pattern. In the cold months, minimum temperature is lower than in the summer, but we also have higher parasite emission when the temperature is lower. This suggests that we should either use julian.date as an explanatory variable or tmin, but not both, as they reflect the same ecological effect (Zuur et al. 2007). Because we want to explicitly model temporal changes, we will use julian.date instead of tmin.

Then, we inspect more closely the bivariate relationships between the response variable and different predictors. Some predictors (testosterone, cortisol, kde90.lag) show unusually high values. Thus, they have been Log_{10}-transformed to down-weight the influence of extreme datapoints.

```
par(mfrow=c(3,3))

plot(log10(data.parasites$testosterone),data.parasites$lungworms,xlab="log10(testosterone)",ylab="LPG")
plot(log10(data.parasites$cortisol),data.parasites$lungworms,xlab="log10(cortisol)",ylab="LPG")
plot(data.parasites$age,data.parasites$lungworms,xlab="age",ylab="LPG")
plot(data.parasites$interactions,data.parasites$lungworms,xlab="interactions",ylab="LPG")
plot(data.parasites$tmin,data.parasites$lungworms,xlab="tmin",ylab="LPG")
```

```
plot(data.parasites$prec,data.parasites$lungworms,xlab="prec",ylab="LPG")
plot(data.parasites$elev.lag,data.parasites$lungworms,xlab="elev.lag",ylab="LPG")
plot(log10(data.parasites$kde90.lag),data.parasites$lungworms,xlab="log10(kde90.lag)",ylab="LPG")
plot(data.parasites$julian.date,data.parasites$lungworms,xlab="julian.date",ylab="LPG")
```

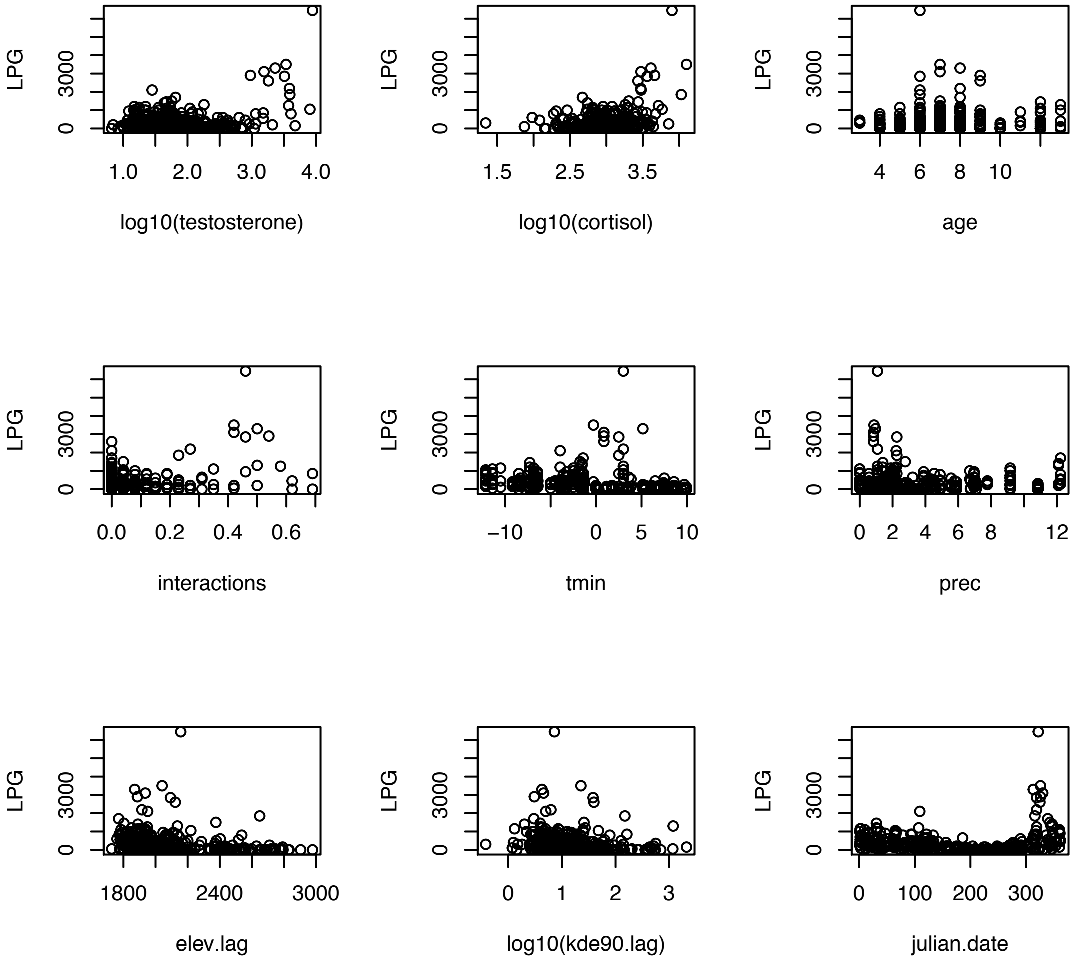

The graphs suggest widespread non-linearity in the relationships between lungworm counts and predictors. We also inspect the tactic-specific variations in all the predictors (Fig. 1).

```
data.parasites$month <- month(data.parasites$POSIX.date) # add month to dataset
head(data.parasites)

# We first need to summarize the variables.
# The following function “summarySE” gives count, mean, standard deviation, standard error of the mean,
# and confidence interval (default 95%).
#   data: a data frame.
#   measurevar: the name of a column that contains the variable to be summariezed
#   groupvars: a vector containing names of columns that contain grouping variables
#   na.rm: a boolean that indicates whether to ignore NA's
#   conf.interval: the percent range of the confidence interval (default is 95%)
summarySE <- function(data=NULL, measurevar, groupvars=NULL, na.rm=FALSE,
                      conf.interval=.95, .drop=TRUE) {
  library(plyr)
  # New version of length which can handle NA's: if na.rm==T, don't count them
  length2 <- function (x, na.rm=FALSE) {
    if (na.rm) sum(!is.na(x))
```

```
    else       length(x)
  }
  # This does the summary. For each group's data frame, return a vector with
  # N, mean, and sd
  datac <- ddply(data, groupvars, .drop=.drop,
                 .fun = function(xx, col) {
                   c(N    = length2(xx[[col]], na.rm=na.rm),
                     mean = mean   (xx[[col]], na.rm=na.rm),
                     sd   = sd     (xx[[col]], na.rm=na.rm)
                   )
                 },
                 measurevar
  )
  # Rename the "mean" column
  datac <- rename(datac, c("mean" = measurevar))
  datac$se <- datac$sd / sqrt(datac$N)  # Calculate standard error of the mean
  # Confidence interval multiplier for standard error
  # Calculate t-statistic for confidence interval:
  # e.g., if conf.interval is .95, use .975 (above/below), and use df=N-1
  ciMult <- qt(conf.interval/2 + .5, datac$N-1)
  datac$ci <- datac$se * ciMult
  return(datac)
}

# summarize variables
lungworms.sum <- summarySE(data.parasites, measurevar="lungworms", groupvars=c("m.beh", "month"))
age.sum <- summarySE(data.parasites, measurevar="age", groupvars=c("m.beh"))
testo.sum <- summarySE(data.parasites, measurevar="testosterone", groupvars=c("m.beh", "month"))
cortisol.sum <- summarySE(data.parasites, measurevar="cortisol", groupvars=c("m.beh", "month"))
interactions.sum <- summarySE(data.parasites, measurevar="interactions", groupvars=c("m.beh", "month"))
elev.lag.sum <- summarySE(data.parasites, measurevar="elev.lag", groupvars=c("m.beh", "month"))
kde90.lag.sum <- summarySE(data.parasites, measurevar="kde90.lag", groupvars=c("m.beh", "month"))
tmin.sum <- summarySE(data.parasites, measurevar="tmin", groupvars=c("month"))
prec.sum <- summarySE(data.parasites, measurevar="prec", groupvars=c("month"))
```

We plot Figure 1.

```
library(ggplot2)
pd <- position_dodge(0.2)

age.plot <- ggplot(age.sum, aes(x=m.beh, y=age, group=1, colour=m.beh)) +
  theme_linedraw() +
  theme(panel.background=element_blank(), panel.grid.major=element_blank(), panel.grid.minor=element_blank()) +
  geom_errorbar(width=.1, aes(ymin=age-ci, ymax=age+ci), colour="black") +
  geom_point(shape=21, size=3, fill="white") +
  annotation_custom(grobTree(textGrob("A", x=0.025,  y=0.95, hjust=0,
                                      gp=gpar(col="black", fontsize=12)))) +
  xlab("Mating behaviour") +
  ylab("Age (in years)") +
  scale_y_continuous(limits = c(6.5, 7.75), breaks=c(6.5, 7.0, 7.5)) +
  theme(legend.position="NULL")

interactions.plot <- ggplot(interactions.sum, aes(x=month, y=interactions, colour=m.beh, group=m.beh)) +
  theme_linedraw() +
  theme(panel.background=element_blank(), panel.grid.major=element_blank(), panel.grid.minor=element_blank()) +
  geom_errorbar(aes(ymin=interactions-se, ymax=interactions+se), colour="black", width=.1, position=pd) +
  geom_line(position=pd) +
  geom_point(position=pd, size=3, shape=21, fill="white") +
  annotation_custom(grobTree(textGrob("B", x=0.025,  y=0.95, hjust=0,
                                      gp=gpar(col="black", fontsize=12)))) +
  xlab("Month") +
  ylab("Interactions (proportion of time)") +
  scale_colour_hue(name="Mating behaviour",
                   breaks=c("T", "NT"),
                   labels=c("Territorial", "Nonterritorial")) +
  scale_x_continuous(limits = c(0.9,12.1), breaks=c(1,2,3,4,5,6,7,8,9,10,11,12)) +
  theme(legend.position=c(0.25, 0.8))
```

```
testo.plot <- ggplot(testo.sum, aes(x=month, y=testosterone, colour=m.beh, group=m.beh)) +
  theme_linedraw() +
  theme(panel.background=element_blank(), panel.grid.major=element_blank(), panel.grid.minor=element_blank()) +
  geom_errorbar(aes(ymin=testosterone-se, ymax=testosterone+se), colour="black", width=.1, position=pd) +
  geom_line(position=pd) +
  geom_point(position=pd, size=3, shape=21, fill="white") +
  annotation_custom(grobTree(textGrob("C", x=0.025,  y=0.95, hjust=0,
                                      gp=gpar(col="black", fontsize=12)))) +
  xlab("Month") +
  ylab("Testosterone metabolites (ng/g)") +
  scale_colour_hue(name="Mating behaviour",
                   breaks=c("T", "NT"),
                   labels=c("Territorial", "Nonterritorial")) +
  scale_x_continuous(limits = c(0.9,12.1), breaks=c(1,2,3,4,5,6,7,8,9,10,11,12)) +
  theme(legend.position=c(0.25, 0.8))

cortisol.plot <- ggplot(cortisol.sum, aes(x=month, y=cortisol, colour=m.beh, group=m.beh)) +
  theme_linedraw() +
  theme(panel.background=element_blank(), panel.grid.major=element_blank(), panel.grid.minor=element_blank()) +
  geom_errorbar(aes(ymin=cortisol-se, ymax=cortisol+se), colour="black", width=.1, position=pd) +
  geom_line(position=pd) +
  geom_point(position=pd, size=3, shape=21, fill="white") +
  annotation_custom(grobTree(textGrob("D", x=0.025,  y=0.95, hjust=0,
                                      gp=gpar(col="black", fontsize=12)))) +
  xlab("Month") +
  ylab("Cortisol metabolites (ng/g)") +
  scale_colour_hue(name="Mating behaviour",
                   breaks=c("T", "NT"),
                   labels=c("Territorial", "Nonterritorial")) +
  scale_x_continuous(limits = c(0.9,12.1), breaks=c(1,2,3,4,5,6,7,8,9,10,11,12)) +
  theme(legend.position=c(0.25, 0.8))

elev.lag.plot <- ggplot(elev.lag.sum, aes(x=month, y=elev.lag, colour=m.beh, group=m.beh)) +
  theme_linedraw() +
  theme(panel.background=element_blank(), panel.grid.major=element_blank(), panel.grid.minor=element_blank()) +
  geom_errorbar(aes(ymin=elev.lag-se, ymax=elev.lag+se), colour="black", width=.1, position=pd) +
  geom_line(position=pd) +
  geom_point(position=pd, size=3, shape=21, fill="white") +
  annotation_custom(grobTree(textGrob("E", x=0.025,  y=0.95, hjust=0,
                                      gp=gpar(col="black", fontsize=12)))) +
  xlab("Month") +
  ylab("1-month lagged elevation (in m)") +
  scale_colour_hue(name="Mating behaviour",
                   breaks=c("T", "NT"),
                   labels=c("Territorial", "Nonterritorial")) +
  scale_x_continuous(limits = c(0.9,12.1), breaks=c(1,2,3,4,5,6,7,8,9,10,11,12)) +
  theme(legend.position=c(0.25, 0.8))


kde90.lag.plot <- ggplot(kde90.lag.sum, aes(x=month, y=kde90.lag, colour=m.beh, group=m.beh)) +
  theme_linedraw() +
  theme(panel.background=element_blank(), panel.grid.major=element_blank(), panel.grid.minor=element_blank()) +
  geom_errorbar(aes(ymin=kde90.lag-se, ymax=kde90.lag+se), colour="black", width=.1, position=pd) +
  geom_line(position=pd) +
  geom_point(position=pd, size=3, shape=21, fill="white") +
  annotation_custom(grobTree(textGrob("F", x=0.025,  y=0.95, hjust=0,
                                      gp=gpar(col="black", fontsize=12)))) +
  xlab("Month") +
  ylab("1-month lagged Home range (in ha)") +
  scale_colour_hue(name="Mating behaviour",
                   breaks=c("T", "NT"),
                   labels=c("Territorial", "Nonterritorial")) +
  scale_x_continuous(limits = c(0.9,12.1), breaks=c(1,2,3,4,5,6,7,8,9,10,11,12)) +
  theme(legend.position=c(0.25, 0.8))

tmin.plot <- ggplot(tmin.sum, aes(x=month, y=tmin, group=1)) +
  theme_linedraw() +
  theme(panel.background=element_blank(), panel.grid.major=element_blank(), panel.grid.minor=element_blank()) +
  geom_errorbar(aes(ymin=tmin-se, ymax=tmin+se), colour="black", width=.1, position=pd) +
  geom_line() +
```

```
  geom_point(size=3, shape=21, fill="white") +
  annotation_custom(grobTree(textGrob("G", x=0.025,  y=0.95, hjust=0,
                                      gp=gpar(col="black", fontsize=12)))) +
  xlab("Month") +
  ylab("20/40-days lagged min. temperature (in °C)") +
  scale_x_continuous(limits = c(0.9,12.1), breaks=c(1,2,3,4,5,6,7,8,9,10,11,12))

prec.plot <- ggplot(prec.sum, aes(x=month, y=prec, group=1)) +
  theme_linedraw() +
  theme(panel.background=element_blank(), panel.grid.major=element_blank(), panel.grid.minor=element_blank()) +
  geom_errorbar(aes(ymin=prec-se, ymax=prec+se), colour="black", width=.1, position=pd) +
  geom_line() +
  geom_point(size=3, shape=21, fill="white") +
  annotation_custom(grobTree(textGrob("H", x=0.025,  y=0.95, hjust=0,
                                      gp=gpar(col="black", fontsize=12)))) +
  xlab("Month") +
  ylab("20/40-days lagged precipitation (in mm)") +
  scale_x_continuous(limits = c(0.9,12.1), breaks=c(1,2,3,4,5,6,7,8,9,10,11,12))

library(gridExtra)
grid.arrange(age.plot, interactions.plot,
             testo.plot, cortisol.plot,
             elev.lag.plot, kde90.lag.plot,
             tmin.plot, prec.plot, nrow=4)
```

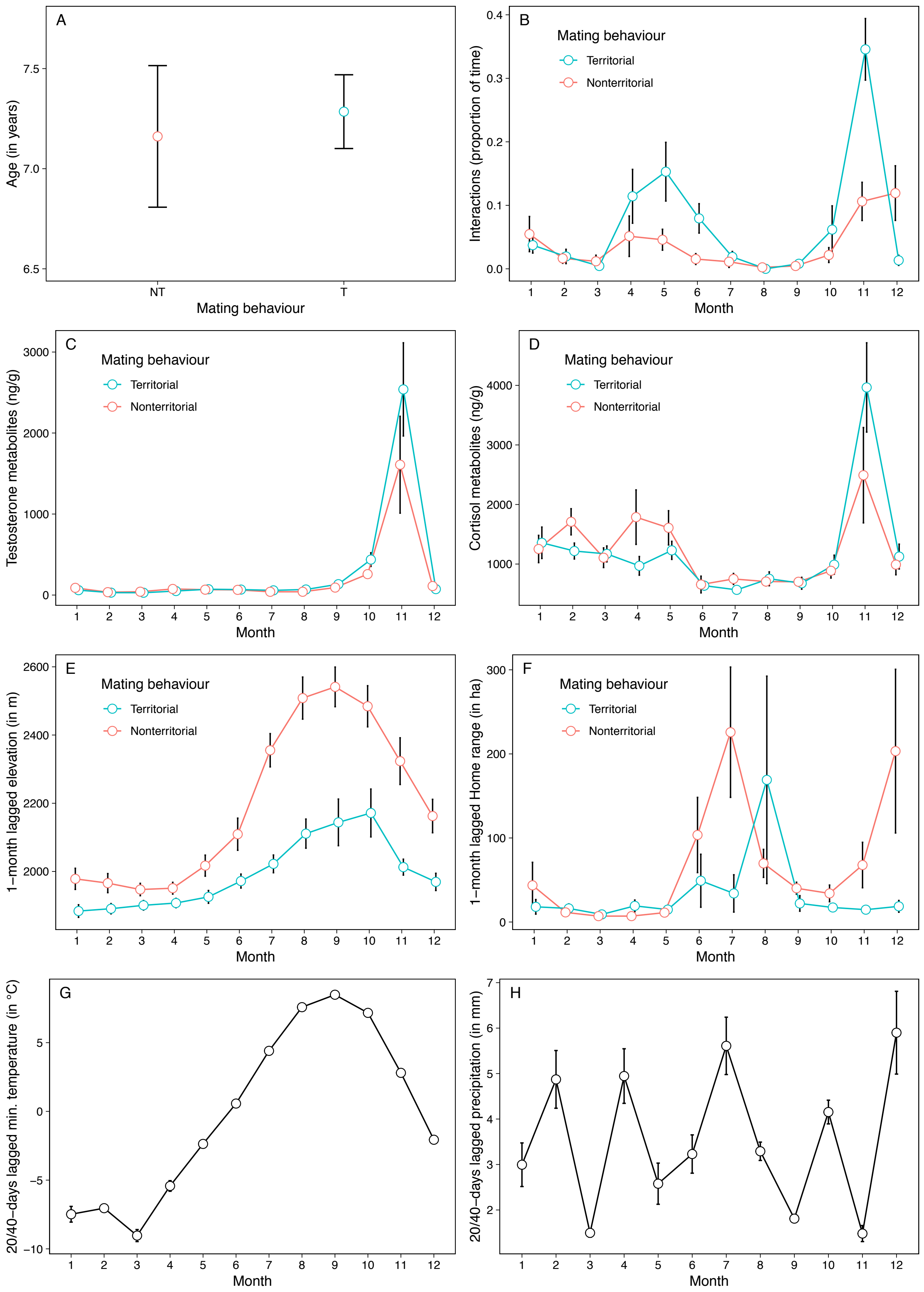

A
Age (in years)
Mating behaviour
NT
T
B
Interactions (proportion of time)
Mating behaviour
Territorial
Nonterritorial
Month
C
Testosterone metabolites (ng/g)
D
Cortisol metabolites (ng/g)
E
1−month lagged elevation (in m)
F
1−month lagged Home range (in ha)
G
20/40−days lagged min. temperature (in °C)
H
20/40−days lagged precipitation (in mm)

Then we plot Figure 2 (the tactic-specific monthly patterns of larvae per gram (LPG) in territorial (T) and nonterritorial (NT) male chamois).

```
lungworms.plot <- ggplot(lungworms.sum, aes(x=month, y=lungworms, colour=m.beh, group=m.beh)) +
  theme_linedraw() +
  theme(panel.background=element_blank(),panel.grid.major=element_blank(),panel.grid.minor=element_blank()) +
  geom_errorbar(aes(ymin=lungworms-se, ymax=lungworms+se), colour="black", width=.1, position=pd) +
  geom_line(position=pd) +
  geom_point(position=pd, size=3, shape=21, fill="white") +
  xlab("Month") +
  ylab("Larvae per gram (LPG)") +
  scale_colour_hue(name="Mating behaviour",
                   breaks=c("T", "NT"),
                   labels=c("Territorial", "Nonterritorial")) +
  scale_x_continuous(limits = c(0.9,12.1), breaks=c(1,2,3,4,5,6,7,8,9,10,11,12)) +
  theme(legend.position=c(0.25, 0.8))

lungworms.plot
```

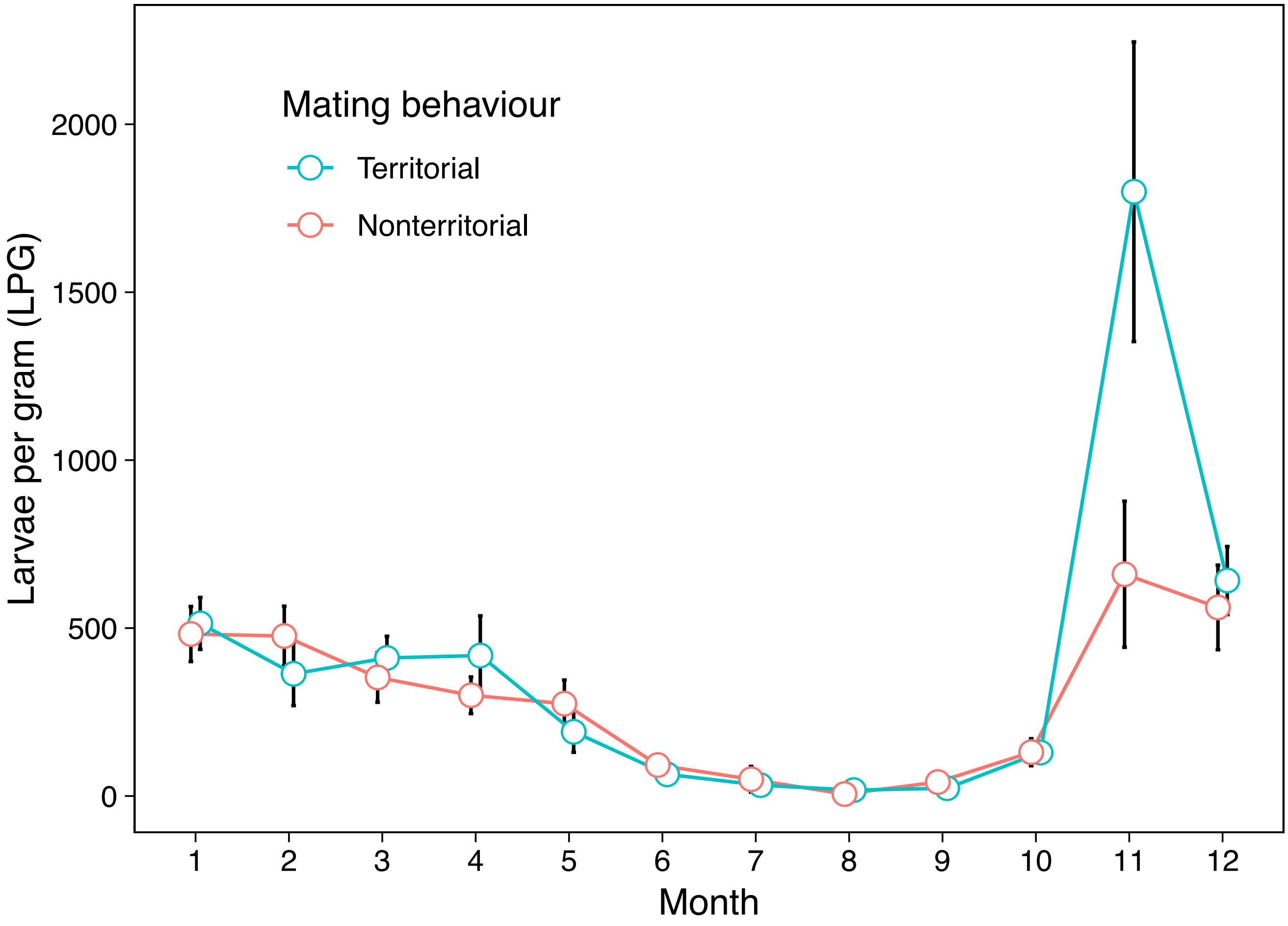

4.2 Overdispersion and zero-inflation

We first fit a Poisson model.

```
library(glmmTMB)
fit <- glmmTMB(lungworms ~ m.beh+scale(age)+scale(interactions)+
                 scale(log10(testosterone))+scale(log10(cortisol))+
                 scale(tmin)+scale(prec)+
                 scale(log10(kde90.lag))+scale(elev.lag)+
                 (1|id),
               data = data.parasites,
               ziformula = ~0,
               family=poisson())

library(sjstats)
overdisp(fit) # overdispersion
zero_count(fit) # difference in zero counts
##
## # Overdispersion test
##
##        dispersion ratio = 261.0702
##   Pearson's Chi-Squared = 99728.8038
##                 p-value = 0.0000
## Overdispersion detected.
##
## # Zero-Count overfitting
##
##    Observed zero-counts: 117
##   Predicted zero-counts: 0
##                   Ratio: 0.00
## Model is underfitting zero-counts (probable zero-inflation).
```

The tests indicate issues of overdispersion and zero-inflation.

5. Modelling

In our model we have to account for the correlation among larval counts that stemmed from multiple sampling of the same individuals over time, and for non-linearity in some predictor-response relationships revealed by exploratory analysis.

Given these issues, and considering the problem of overdispersion and zero-inflation, the change in larval counts was investigated fitting a generalized additive mixed model (GAMM), using the package 'gamlss'. We assumed a negative binomial distribution and allowed for zero-inflation (i.e., zero-inflated negative binomial distribution).

In all models, the method used for selecting the smoother was "ML".

```
library(gamlss)
```

5.1 Model fitting

We start by fitting a 'null' GAMM, i.e., we model larval counts simply as a function of Julian date grouped by mating behavior (T *vs.* NT). This model can be thought of as the 'pure' relationship between larval count and Julian date in ARTs.

In the 'null' model, the number of equally spaced interval between the minimum and the maximum values of x used for the creation of splines was reduced to 6.55, as the default number (20) appears to overfit. 'inter = 6.55' returned the lowest value for the Bayesian Criterion (SBC = 4378.491).

```
mod.null.lungworms<-gamlss(lungworms ~ pvc(julian.date,by=m.beh,inter=6.55) +
                             random(id),
                           data=data.parasites,
                           family=ZINBI,
                           method=CG(300))
```

We then model larval counts by fitting a 'global' GAMM that includes all the available variables, except tmin.

```
mod.tot.lungworms<-gamlss(lungworms ~ pvc(julian.date,by=m.beh) +
                            pb(age) + pb(interactions) +
                            pb(log10(testosterone)) + pb(log10(cortisol)) +
                            pb(log10(kde90.lag)) + pb(elev.lag) +
                            pb(prec) +
                            random(id),
                          data=data.parasites,
                          family=ZINBI,
                          method=CG(1000),normalize=TRUE)
```

We perform a model selection on the 'global' model using an AIC-based stepwise procedure, to obtain a 'final' GAMM.

```
mod.selection.lungworms <- stepGAIC(mod.tot.lungworms)
```

We re-fit the 'final' model selected through stepwise.

This model can be thought of as the 'leftover' effect of Julian date after accounting for the explanatory effect of the selected predictors.

```
mod.final.lungworms<-gamlss(lungworms ~ pvc(julian.date,by=m.beh) +
                              pb(age) + pb(interactions) +
                              pb(log10(testosterone)) + pb(log10(cortisol)) +
                              pb(prec) +
                              random(id),
                            data=data.parasites,
                            family=ZINBI,
                            method=CG(300))

summary(mod.final.lungworms)

## ******************************************************************
## Family:  c("ZINBI", "Zero inflated negative binomial type I")
##
## Call:  gamlss(formula = lungworms ~ pvc(julian.date, by = m.beh) +
##     pb(age) + pb(interactions) + pb(log10(testosterone)) +
##     pb(log10(cortisol)) + pb(prec) + random(id), family = ZINBI,
##     data = data.parasites, method = CG(300))
##
## Fitting method: CG(300)
##
## ------------------------------------------------------------------
## Mu link function:  log
## Mu Coefficients:
##                         Estimate Std. Error t value Pr(>|t|)
## (Intercept)             -2.81009    0.35953  -7.816 6.46e-14 ***
## pb(age)                  0.14767    0.01870   7.898 3.71e-14 ***
## pb(interactions)             0.30777    0.33914   0.908    0.365
## pb(log10(testosterone))  0.82988    0.07499  11.066  < 2e-16 ***
## pb(log10(cortisol))      1.94965    0.11438  17.045  < 2e-16 ***
## pb(prec)                 0.05981    0.01135   5.269 2.41e-07 ***
## ---
## Signif. codes:  0 '***' 0.001 '**' 0.01 '*' 0.05 '.' 0.1 ' ' 1
##
## ------------------------------------------------------------------
## Sigma link function:  log
## Sigma Coefficients:
##             Estimate Std. Error t value Pr(>|t|)
## (Intercept) -1.04501    0.08147  -12.83   <2e-16 ***
## ---
## Signif. codes:  0 '***' 0.001 '**' 0.01 '*' 0.05 '.' 0.1 ' ' 1
##
##
##
##
##
```

```
## ------------------------------------------------------------------
## Nu link function:  logit 
## Nu Coefficients:
##             Estimate Std. Error t value Pr(>|t|)    
## (Intercept)  -0.8579     0.1103  -7.776 8.46e-14 ***
## ---
## Signif. codes:  0 '***' 0.001 '**' 0.01 '*' 0.05 '.' 0.1 ' ' 1
## 
## ------------------------------------------------------------------
## NOTE: Additive smoothing terms exist in the formulas: 
##  i) Std. Error for smoothers are for the linear effect only. 
## ii) Std. Error for the linear terms maybe are not accurate. 
## ------------------------------------------------------------------
## No. of observations in the fit:  393 
## Degrees of Freedom for the fit:  43.24886
##       Residual Deg. of Freedom:  349.7511 
##                       at cycle:  96 
##  
## Global Deviance:     4093.103 
##             AIC:     4179.601 
##             SBC:     4351.463 
## ******************************************************************
```

We adjust the significance level for the maximum number of steps in the stepwise selection (Bonferroni correction).

```
p_value_threshold <- 0.05 / factorial(9)
p_value_threshold
## [1] 1.377866e-07
```

Faecal larval counts were positively related to hormone metabolites through non-linear relationships, and to age through a linear relationship. Interactions and precipitation were not significant.

We calculate the pseudo-R^2 of the final model.

```
Rsq(mod.final.lungworms)
## [1] 0.57
```

We also calculate the pseudo-R^2 (Nagelkerke's generalized R-squared) of the model fitted without random term, to explore the improvement of the model's fit when the random term is included.

```
mod.final.lungworms.no.random <-gamlss(lungworms ~ pvc(julian.date,by=m.beh) + 
                                pb(age) + pb(interactions) + 
                                pb(log10(testosterone)) + pb(log10(cortisol)) + 
                                pb(prec)),
                              data=data.parasites,
                              family=ZINBI,
                              method=CG(300))
Rsq(mod.final.lungworms.no.random)
## [1] 0.45
```

5.2 Model diagnostic

We check the diagnostics of the 'null' and the 'final' models using plots of residuals against predicted values, density estimates of residuals, normal qq-plot, summaries of the randomized quantile residuals, and wormplots.

Null model

```
par(mfrow=c(1,3))
plot(predict(mod.null.lungworms),resid(mod.null.lungworms),xlab="Predicted",ylab="Residuals",main="Against Predic
ted Values")
plot(density(resid(mod.final.lungworms)),main="Density Estimate",xlab="Quantile residuals")
qqnorm(resid(mod.final.lungworms))
```

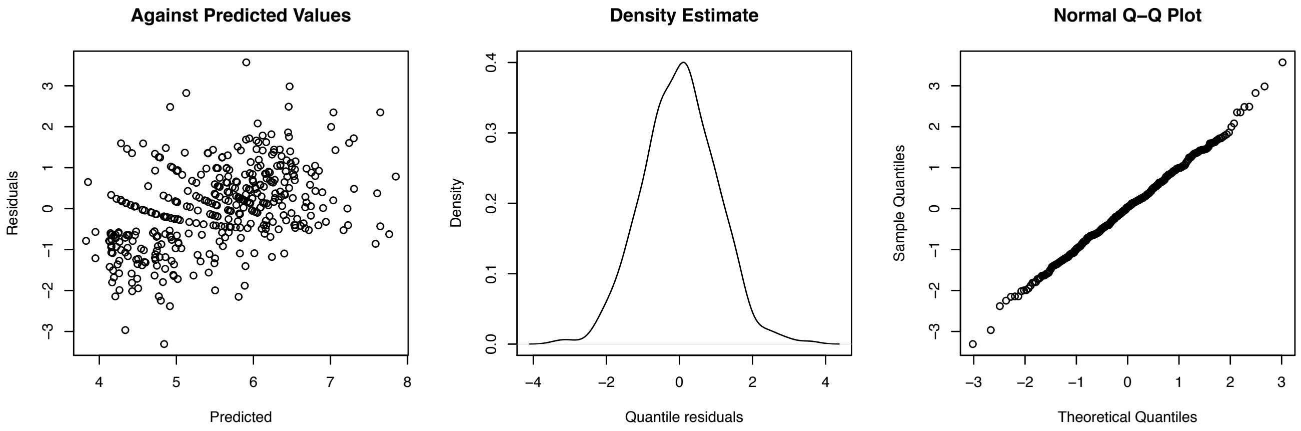

The plots above appear to suggest reasonable behavior, albeit with some area of misfit. We thus have a look at the summary statistics.

```
plot(mod.null.lungworms)

******************************************************************
         Summary of the Randomised Quantile Residuals
                              mean   =  0.007759012
                          variance   =  1.009343
                coef. of skewness  =  0.05218517
                coef. of kurtosis  =  3.306742
Filliben correlation coefficient  =  0.9985132
******************************************************************
```

The summary statistics for the quantile residuals shows that the mean is nearly zero, the variance is nearly 1, the coefficient of skewness is near 0, and the coefficient of kurtosis is near 3. The statistics therefore suggest that the residuals are approximately normally distributed. Furthermore, the Filliben coefficient of correlation (i.e., the normal probability plot correlation coefficient) is near 1.

As a final model diagnostic tool, we use a wormplot. In gamlss, when the distribution of the response variable is discrete, it is advisable to use the function rqres.plot, which creates multiple realizations of the normalized randomized quantile residuals (Stasinopoulos et al. 2017).

```
rqres.plot(mod.null.lungworms, ylim.all=TRUE)
```

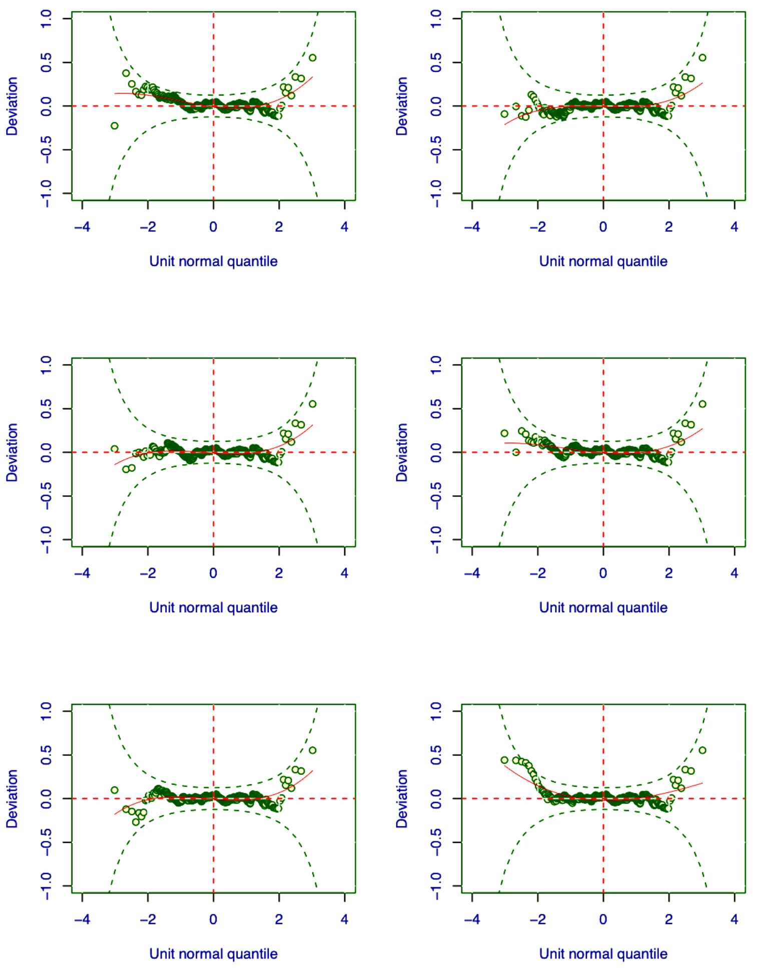

All six realizations show reasonable behavior.

Final model

```
par(mfrow=c(1,3))
plot(predict(mod.final.lungworms),resid(mod.final.lungworms),xlab="Predicted",ylab="Residuals",main="Against Pred
icted Values")
plot(density(resid(mod.final.lungworms)),main="Density Estimate",xlab="Quantile residuals")
qqnorm(resid(mod.final.lungworms))
```

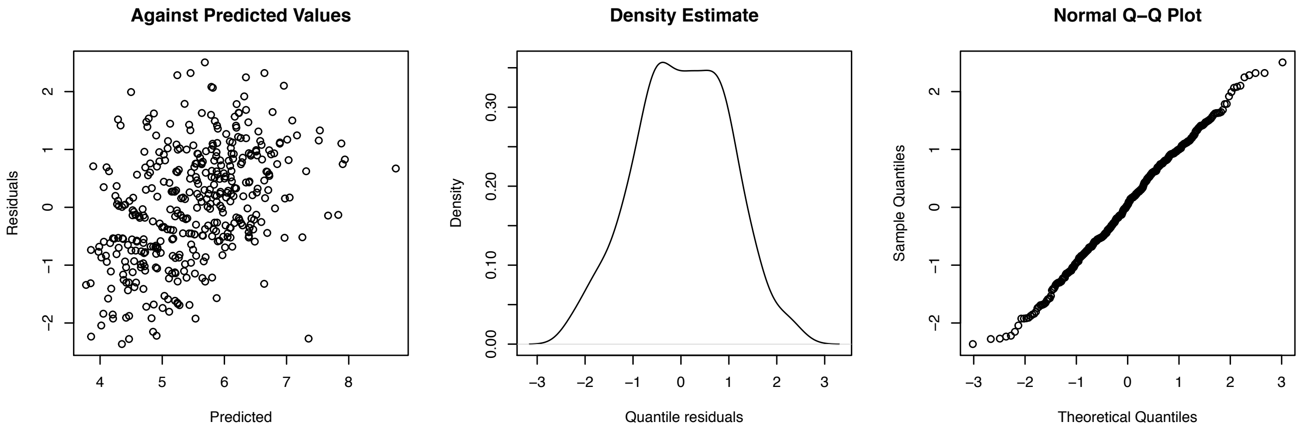

The plots above appear to suggest reasonable behavior, albeit with some area of misfit, possibly related to issues of kurtosis/skewness due to extreme values? We thus have a look at the summary statistics.

```
plot(mod.final.lungworms)

******************************************************************
         Summary of the Randomised Quantile Residuals
                           mean   =  0.007155068
                       variance   =  1.010711
               coef. of skewness  =  -0.1667212
               coef. of kurtosis  =  2.727381
Filliben correlation coefficient  =  0.9976762
******************************************************************
```

The summary statistics for the quantile residuals shows that the mean is nearly zero, the variance is nearly 1, the coefficient of skewness is near 0, and the coefficient of kurtosis is near 3. The statistics therefore suggest that the residuals are approximately normally distributed. Furthermore, the Filliben coefficient of correlation (i.e., the normal probability plot correlation coefficient) is near 1.

As a final model diagnostic tool, we use a wormplot. In gamlss, when the distribution of the response variable is discrete, it is advisable to use the function rqres.plot, which creates multiple realizations of the normalized randomized quantile residuals (Stasinopoulos et al. 2017).

```
rqres.plot(mod.final.lungworms, ylim.all=TRUE)
```

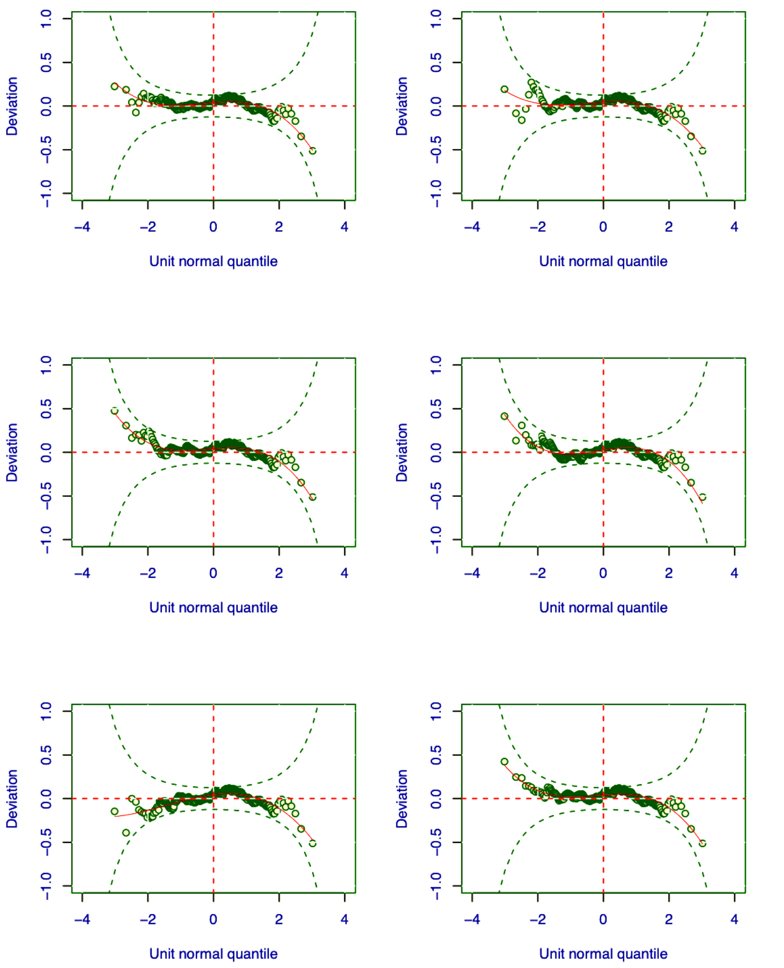

All six realizations show reasonable behavior.

6. Plot model results

We plot the results of the 'null' and 'final' models. First, we need to extract temporal fitted values (mean and variance) from both models. Then we calculate the 95% confidence interval and include the mean fitted values and confidence intervals in the original dataset. We repeat the procedure for the "null" and the "final" model.

```
fit.null.lungworms <- as.data.frame(mod.null.lungworms$mu.s)
var.null.lungworms <- as.data.frame(mod.null.lungworms$mu.var)
data.parasites$jd.beh.null.lungworms<-fit.null.lungworms$`pvc(julian.date,by = m.beh,inter = 6.55)`
data.parasites$jd.beh.up.ci.null.lungworms<-data.parasites$jd.beh.null.lungworms+
                                                                 1.96*(sqrt((var.null.lungworms$`pvc(julian.date,by=m.beh,inter=6.55)`)))
data.parasites$jd.beh.lw.ci.null.lungworms<-data.parasites$jd.beh.null.lungworms-
                                                                 1.96*(sqrt((var.null.lungworms$`pvc(julian.date,by=m.beh,inter=6.55)`)))
```

```
fit.final.lungworms <- as.data.frame(mod.final.lungworms$mu.s)
var.final.lungworms <- as.data.frame(mod.final.lungworms$mu.var)
data.parasites$jd.beh.final.lungworms<-fit.final.lungworms$`pvc(julian.date, by = m.beh)`
data.parasites$jd.beh.up.ci.final.lungworms<-data.parasites$jd.beh.final.lungworms+
                                             1.96*(sqrt((var.final.lungworms$`pvc(julian.date,by=m.beh)`)))
data.parasites$jd.beh.lw.ci.final.lungworms<-data.parasites$jd.beh.final.lungworms-
                                             1.96*(sqrt((var.final.lungworms$`pvc(julian.date,by=m.beh)`)))
```

6.1 Figure 3

The figure shows the relationship between Julian date and faecal larval counts in territorial (T) and nonterritorial (NT) male chamois. In A, plain ('pure') effect of Julian date on larvae per gram. In B, 'leftover' effect of Julian date after controlling for the explanatory effects of testosterone and cortisol metabolites, age, interactions and precipitation. Vertical lines indicate the onset of territoriality (April to mid-May) and the rutting period (early November – early December).

```
library(ggplot2)

null.model.effect.lungworms <- ggplot(data.parasites,aes(x=julian.date,y=jd.beh.null.lungworms,col=m.beh)) +
  theme_bw() +
  theme(panel.grid.major=element_blank(),panel.grid.minor=element_blank()) +
  coord_cartesian(xlim=c(0,365),ylim=c(-2.5,2.5)) +
  geom_ribbon(aes(ymin=jd.beh.lw.ci.null.lungworms,ymax=jd.beh.up.ci.null.lungworms,fill=m.beh),
                                                                                   alpha=0.50,colour=NA) +
  scale_fill_manual(values=c("blue","green")) +
  scale_color_manual(values=c(T="black",NT="black")) +
  geom_line(aes(julian.date,jd.beh.null.lungworms,linetype=m.beh)) +
  geom_vline(xintercept=c(90,135,310,340), linetype=1, col="red", cex=0.25) +
  xlab("Julian date") +
  ylab("Partial for pvc(Julian date,by=Mating behaviour)") +
  scale_x_continuous(limits=c(0,365),breaks=c(0,50,100,150,200,250,300,350)) +
  scale_y_continuous(limits=c(-2.5,2.5),breaks=c(-2,-1,0,1,2)) +
  annotate("text",x=0,y=2.4,label="A",color="black",angle=0) +
  annotate("text",x=112, y = -1.75, label = "Territoriality onset", color="red", angle = 90) +
  annotate("text",x=325, y = -1.75, label = "Rut", color="red", angle = 90) +
  theme(legend.position="right") +
  theme(legend.title = element_blank())

final.model.effect.lungworms <- ggplot(data.parasites,aes(x=julian.date,y=jd.beh. final.lungworms,col=m.beh)) +
  theme_bw() +
  theme(panel.grid.major=element_blank(),panel.grid.minor=element_blank()) +
  coord_cartesian(xlim=c(0,365),ylim=c(-2.5,2.5)) +
  geom_ribbon(aes(ymin=jd.beh.lw.ci.final.lungworms,ymax=jd.beh.up.ci.final.lungworms,fill=m.beh),
                                                                                   alpha=0.50,colour=NA) +
  scale_fill_manual(values=c("blue","green")) +
  scale_color_manual(values=c(T="black",NT="black")) +
  geom_line(aes(julian.date,jd.beh.final.lungworms,linetype=m.beh)) +
  geom_vline(xintercept=c(90,135,310,340), linetype=1, col="red", cex=0.25) +
  xlab("Julian date") +
  ylab("Partial for pvc(Julian date,by=Mating behaviour)") +
  scale_x_continuous(limits=c(0,365),breaks=c(0,50,100,150,200,250,300,350)) +
  scale_y_continuous(limits=c(-2.5,2.5),breaks=c(-2,-1,0,1,2)) +
  annotate("text",x=0,y=2.4,label="A",color="black",angle=0) +
  annotate("text",x=112, y = -1.75, label = "Territoriality onset", color="red", angle = 90) +
  annotate("text",x=325, y = -1.75, label = "Rut", color="red", angle = 90) +
  theme(legend.position="right") +
  theme(legend.title = element_blank())

library(gridExtra)
grid.arrange(null.model.effect.lungworms, final.model.effect.lungworms, nrow=1)
```

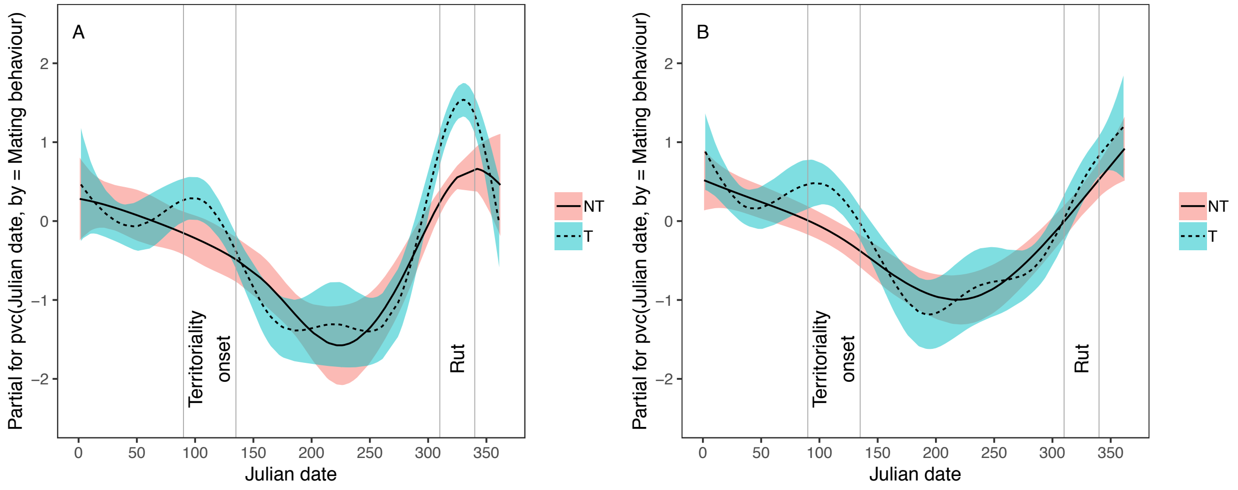

6.2 Figure 4

The figure shows the relationships between faecal larval counts and (A) testosterone, (B) cortisol and (C) age in male chamois.

```
par(mfrow=c(1,3))
par(las=1)
# testosterone
termplot(mod.final.lungworms, se = TRUE, terms = "pb(log10(testosterone))",
         rug=TRUE, col.term = "black", lwd.term = 2,ylim = c(-2,2),
         col.se = "black",lty.se = 2, lwd.se = 1,cex = 1,pch = par("pch"),
         span.smth = 2/3,xlabs = "Testosterone (ng/g)", ylabs = "Partial for pb(Testosterone)", main = NULL)
text(0, 1.75,"A")
# cortisol
termplot(mod.final.lungworms, se = TRUE, terms = "pb(log10(cortisol))",
         rug=TRUE, col.term = "black", lwd.term = 2,ylim = c(-2,2),
         col.se = "black",lty.se = 2, lwd.se = 1,cex = 1,pch = par("pch"),
         span.smth = 2/3,xlabs = "Cortisol (ng/g)", ylabs = "Partial for pb(Cortisol)", main = NULL)
text(0, 1.75,"B")
# age
termplot(mod.final.lungworms, se = TRUE, terms = "pb(age)",
         rug=TRUE, col.term = "black", lwd.term = 2,ylim = c(-2,2),
         col.se = "black",lty.se = 2, lwd.se = 1,cex = 1,pch = par("pch"),
         span.smth = 2/3,xlabs = "Age (years)", ylabs = "Partial for pb(Age)", main = NULL)
text(3, 1.75,"C")
```

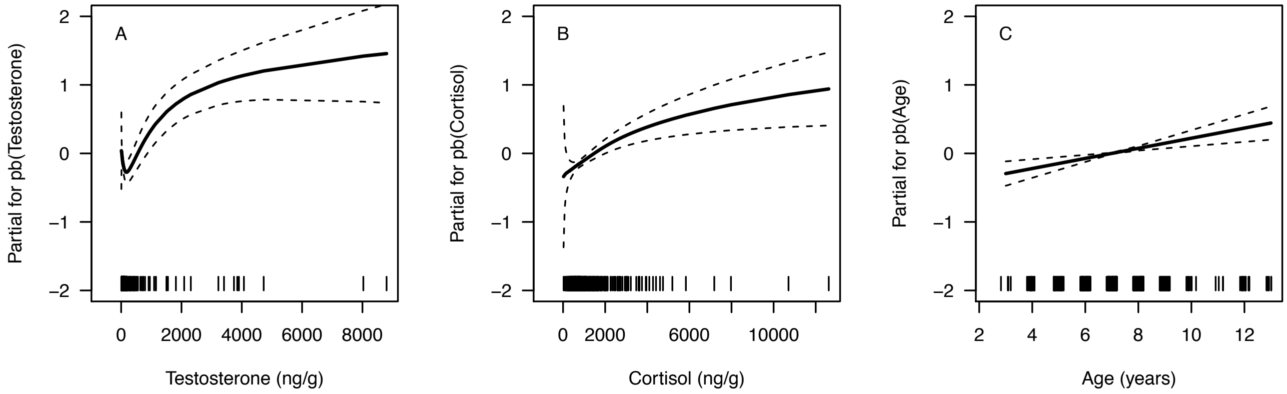

References

- Everitt, B.S., Landau, S., Leese, M., Stahl, D. (2011). *Cluster Analysis*. Wiley.
- Mahalanobis, P.C. (1936). On the generalised distance in statistics. Proceedings of the National Institute of Sciences of India, 2, 49-55.
- Stasinopoulos, M.D., Rigby, R.A., Heller, G.Z., Voudouris, V., De Bastiani, F. (2017). *Flexible Regression and Smoothing – Using GAMLSS in R*. CRC Press.
- Zuur, A.F., Ieno, E.N., Smith, G.M. (2007). *Analysing Ecological Data*. Springer.
